# Correlation between diabetic retinopathy and diabetic nephropathy: a two-sample Mendelian randomization study

**DOI:** 10.3389/fendo.2023.1265711

**Published:** 2023-11-01

**Authors:** Jiaxi Fang, Chuxuan Luo, Di Zhang, Qiang He, Lin Liu

**Affiliations:** ^1^Urology & Nephrology Center, Department of Nephrology, Zhejiang Provincial People’s Hospital (Affiliated People’s Hospital, Hangzhou Medical College), Hangzhou, Zhejiang, China; ^2^Department of Ultrasound, Taizhou Central Hospital (Taizhou University, Hospital), Taizhou, Zhejiang, China; ^3^Department of Nephrology, Taizhou Hospital of Zhejiang Province, Wenzhou Medical University, Taizhou, Zhejiang, China; ^4^Department of Nephrology, The First Affiliated Hospital of Zhejiang Chinese Medical University (Zhejiang Provincial Hospital of Traditional Chinese Medicine), Hangzhou, Zhejiang, China; ^5^Life Sciences Institute, Zhejiang University, Hangzhou, Zhejiang, China

**Keywords:** Mendelian randomization, diabetic retinopathy, diabetic nephropathy, causality, proliferative diabetic retinopathy

## Abstract

**Rationale & objective:**

A causal relationship concerning diabetic retinopathy (DR) and diabetic nephropathy (DN) has been studied in many epidemiological observational studies. We conducted a two-sample mendelian randomization study from the perspective of genetics to assess these associations.

**Methods:**

20 independent single nucleotide polymorphisms (SNPs) associated with diabetic retinopathy were selected from the FinnGen consortium. Summary-level data for diabetic nephropathy were obtained from the publicly available genome-wide association studies (GWAS) database, FinnGen and CKDGen consortium. Inverse variance weighted (IVW) was selected as the primary analysis. MR-Egger, weighted median (WM), simple mode and weighted mode were used as complementary methods to examine causality. Additionally, sensitivity analyses including Cochran’s Q test, MR-Egger, MR-Pleiotropy Residual Sum and Outlier (MR-PRESSO), and leave-one-out analyses were conducted to guarantee the accuracy and robustness of our MR analysis.

**Results:**

Our current study demonstrated positive associations of genetically predicted diabetic retinopathy with diabetic nephropathy (OR=1.32; P=3.72E-11), type 1 diabetes with renal complications (OR=1.96; P= 7.11E-11), and type 2 diabetes with renal complications (OR=1.26, P=3.58E-04). Further subtype analysis and multivariate mendelian randomization (MVMR) also reached the same conclusion. A significant casualty with DN was demonstrated both in non-proliferative DR (OR=1.07, P=0.000396) and proliferative DR (OR=1.67, P=3.699068E-14). All the findings were robust across several sensitivity analyses.

**Conclusion:**

Consistent with previous clinical studies, our findings revealed a positive correlation between DR and DN, providing genetic evidence for the non-invasive nature of DR in predicting DN.

## Introduction

Chronic kidney disease (CKD) is an irreversible change in renal structure and function caused by various causes, which can last for months or years. Recent studies on disease burden have revealed a significant global rise of 29.3% in all-age prevalence of CKD since 1990, contributing to 35.8 million disability-adjusted life years (DALYs) (1). Among these cases, diabetic nephropathy (DN) accounts for nearly one-third of DALYs, establishing CKD as a major worldwide public health concern (2). Diabetic nephropathy, a severe microvascular complication of diabetes, constitutes the primary cause of both CKD and end-stage renal disease (ESRD). Renal biopsy serves as the gold standard for diagnosing DN, providing valuable treatment guidance and prognostic indicators(3). However, renal biopsy is an invasive procedure, for patients with contraindications to not do, such as renal pyknosis, bleeding tendency, isolated kidney, uncontrolled hypertension, or severe anemia, should not be so as a way to monitor disease (4). In clinical practice, patients with type 2 diabetes often present with proteinuria, with or without DR. If a renal biopsy is not actively performed in such cases and treatment is solely based on diabetic kidney disease (DKD), it often delays the timing of intervention and leads to irreversible outcomes. So, it is crucial to develop noninvasive or minimally invasive and objective approaches to pathological assessment for the diagnosis and treatment of DN.

Diabetic retinopathy (DR) is a microvascular complication of diabetes mellitus that damages the endothelial cells of the retinal capillaries and causes abnormal vascular permeability, ischemia, and neovascularization. In clinical practice, it is usually divided into proliferative diabetic retinopathy and non-proliferative diabetic retinopathy according to the presence of retinal neovascularization (5), which can be diagnosed by ophthalmoscopy that is simpler and more non-invasive than renal biopsy. DR is the leading cause of blindness in adults aged 20 to 74, and almost all type 1 diabetes cases and 60% of type 2 diabetes cases will develop DR after 20 years (6).

The kidney bears a striking resemblance to the eye in terms of origin, development, and structure (7). For example, both the glomeruli and choroid have extensive vascular networks of similar structures; the inner retina and the glomerular filtration barrier share similar developmental pathways, and renin-angiotensin-aldosterone cascades have been found in both the kidney and retina (8). Park et al. also reported that about 26.7% of diabetic patients had both DN and DR (9). A plethora of investigations have substantiated the convergence between DN and DR in terms of their pathogenic mechanisms, as well as the similarities observed during their respective onset and progression (10–12). Glycation end products, a distinct set of compounds, play a pivotal role in this context. The process of non-enzymatic glycosylation, facilitated by hyperglycemia, expedites the generation of these compounds through protein reactions, consequently inducing escalated intracellular oxidative stress. Activation of NF-κB and free radicals can directly impact lipids and proteins, provoking vascular cellular impairment and inciting inflammation, ultimately culminating in renal and fundus vascular dysfunction (13, 14). However, it has been observed that 30% of DN patients with coexisting DR exhibit grade III or IV glomerulopathy, implying a correlation between the severity of glomerulopathy and the presence or absence of DR (15). Jia et al. conducted a national cross-sectional study in 2016 to investigate the prevalence of high levels of proteinuria in patients with T2DM and DR. Specifically, 47.8% of the 3,301 patients included had high levels of proteinuria, with the incidence of DR increasing as urine albumin levels rose (16).

The complications of diabetes presented a tendency of familial clustering. Several genes are shared between ocular and renal organogenesis, including Pax2, BMP7, and WT-1, and disruptions in these genes can give rise to a spectrum of diseases that manifest concomitant ocular and renal involvement (17). Genome-wide association study (GWAS)assume a pivotal role in identifying genes and pathways associated with diabetes complications. For example, porta et al. conducted a GWAS study of 3546 type 1 diabetic patients, resulting in the extraction of data of two thiamine transporter proteins (SLC19A2/3) and their transcription factors (SP1/2) to explore their association with severe retinopathy or nephropathy. They found a reduced incidence of severe retinopathy and a reduced combined phenotype of severe retinopathy and end-stage renal disease in a state of strong chain disequilibrium (18).

With the continuous advancement of sequencing technology, GWAS is widely used to study the genetic mechanism of complex diseases and identify a large number of genetic variants that are significantly associated with diabetes complications, such as diabetic nephropathy, diabetic retinopathy, diabetic cardiopathy, and diabetic painful neuropathy (19–21). Mendelian Randomization (MR) serves as an epidemiological analytical approach that utilizes genetic variations, specifically single nucleotide polymorphisms (SNPs), associated with exposure factors to elucidate the causal relationship between these exposures and subsequent outcomes. By virtue of the random assignment of alleles during conception, MR effectively mitigates biases encountered in clinical studies investigating disease etiology, including unmeasured and unknown confounding factors, as well as reverse causation (22, 23). In this study, we employed two-sample Mendelian randomization to investigate the causal relationship between diabetic retinopathy and diabetic nephropathy, subsequently estimating and comparing the magnitude of association across distinct stages of both conditions. Finally, multivariate Mendelian randomization analysis was conducted to adjust for potential confounding factors, including fasting glucose and HbA1c levels.

## Materials and methods

### Study design

**Figure 1:**
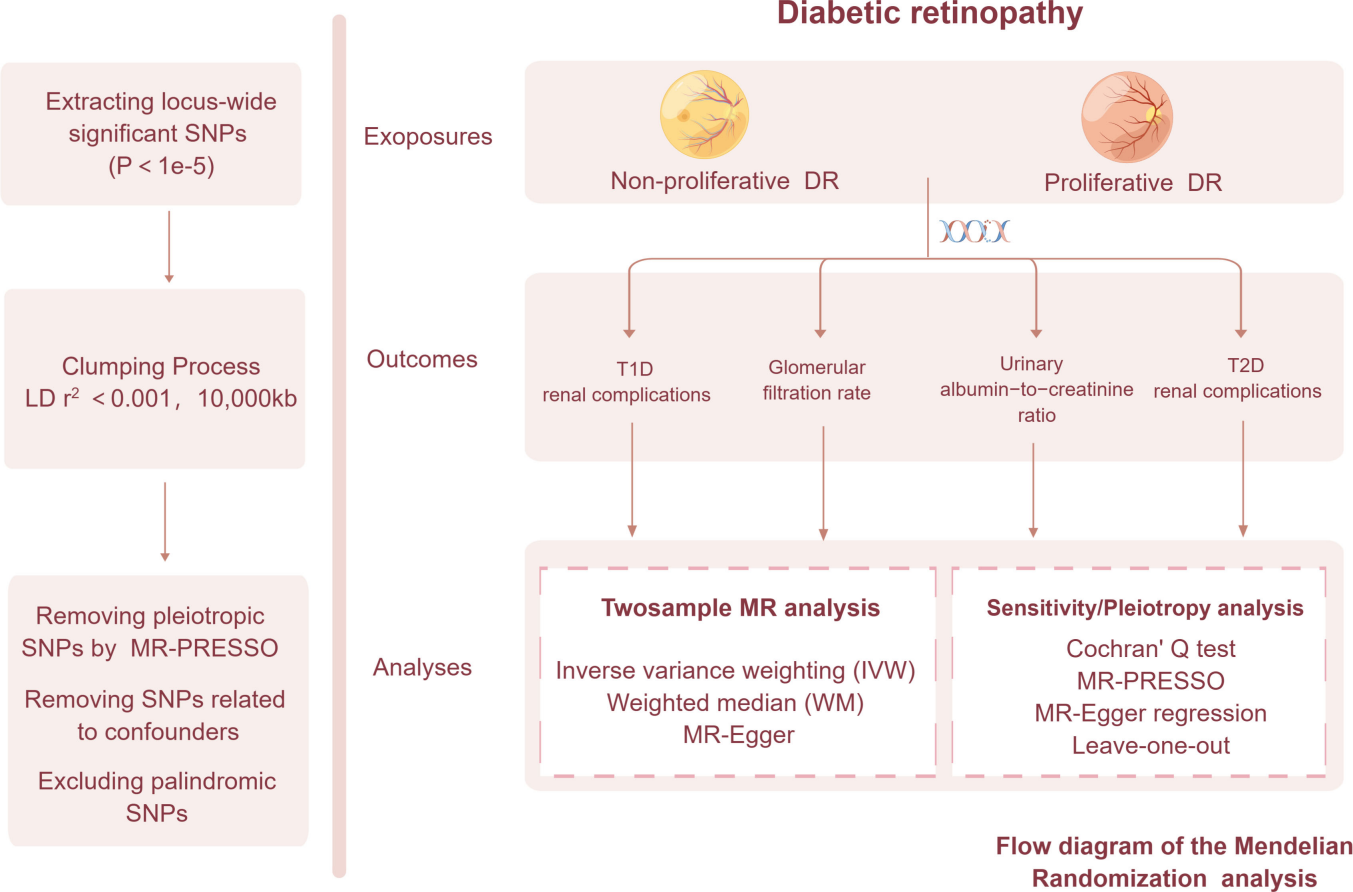
The framework of the Mendelian randomization analysis.

GWAS data pertaining to various stages of diabetic retinopathy and five distinct outcomes associated with diabetic nephropathy were obtained from the publicly accessible GWAS catalog, as well as the CKDGen Consortium (http://ckdgen.imbi.uni-freiburg.de/) and the FinnGen database (www.finngen.fi/fi). Given the re-analysis of previously summarized data, there was no need for additional ethical approval. Comprehensive information regarding the GWAS incorporated in the Mendelian randomization analysis is outlined in **Table 1**. Employing a two-sample Mendelian randomization approach, we initially ascertained the causal association between diabetic retinopathy and five related outcomes associated with diabetic nephropathy. Subsequently, we conducted an in-depth investigation to examine the dynamics of this relationship across distinct stages of diabetic retinopathy, specifically Non-proliferative and Proliferative diabetic retinopathy. Ultimately, potential associations with confounders were subsequently followed-up by multivariate mendelian randomization (MVMR) analyses to investigate the robustness of these associations to adjustment for fasting glucose and HbA1c. Summary statistics of hyperglycemia-related traits, encompassing fasting glucose and HbA1c, were sourced from the Meta-analyses of Glucose and Insulin-Related Traits Consortium (MAGIC), recognized as the most extensive publicly accessible meta-analytic endeavor conducted within non-diabetic populations (https://magicinvestigators.org). The omission of glycemic traits derived from diabetic patients stemmed from our recognition that the influence of anti-diabetic medications or exogenous insulin extends beyond genetic determinants, necessitating their exclusion from our analysis. The MR study relies on three fundamental assumptions (22, 24): (1) The selection of instrumental variables (IVs) must exhibit a significant and robust association with the exposure; (2) The selected instrumental variables (IVs) should be independent of any potential confounding factors that may influence the outcome.; and (3) The selected instrumental variables (IVs) exert a direct effect on the outcome through the exposure variable, without involvement of alternative pathways. The manuscript was meticulously prepared in accordance with the guidelines outlined in the STrengthening the Reporting of Observational Studies in Epidemiology-MR (STROBE-MR) checklist, specifically tailored for a comprehensive two-sample Mendelian randomization (MR) study (25). The schematic representation of our study’s overall workflow is visually presented in **Figure 1**. For the execution of two-sample MR and MVMR analyses, we employed R software (version 4.2.1) along with the Two-sample MR package (version 0.5.5).

**Table 1:** Details of the GWAS studies included in the Mendelian randomization.

Trait	Population	Sample size	Web source
Background diabetic retinopathy	European	206,234	www.finngen.fi/fi
Diabetic nephropathy	European	213,746	www.finngen.fi/fi
Glomerular filtration rate in diabetics	European	144,935	DOI: 10.1038/ncomms10023 PMID: 26831199
Type2 diabetes with renal complications	European	184,481	www.finngen.fi/fi
Type 1 diabetes with renal complications	European	184,148	www.finngen.fi/fi
Urinary albumin-to-creatinine ratio in diabetes	European	51,886	http://ckdgen.imbi.uni-freiburg.de/ PMID: 26631737
Non-proliferative background diabetic retinopathy	European	204,663	www.finngen.fi/fi
Proliferative background diabetic retinopathy	European	212,889	www.finngen.fi/fi
Fasting glucose	European	133,010	MAGIC PMID: 22885924
HbA1c	European	123,665	MAGIC PMID: 20858683

HbA1c, hemoglobin A1c; MAGIC, Meta-analyses of Glucose and Insulin-Related Traits Consortium.

### Genetic instrument selection

Firstly, to identify SNPs that are highly related to diabetic retinopathy, we established the genome-wide significance criterion at *p*<5*10^-8^. Since relatively few SNPs were found for diabetic retinopathy when we constituted the exposure, a looser threshold (*p*<1*10^-5^) was selected (26). Secondly, considering the potential bias caused by solid linkage disequilibrium (LD), we clumped SNPs with LDr^2^< 0.001, kb = 10,000. Palindromic SNPs were excluded because it was not possible to determine if they were aligned for exposure and outcome in the GWASs of diabetic retinopathy. Subsequently, to avoid weak instrument bias, F statistics were calculated to assess instrument strength and the fraction of exposure variance was determined using the R^2^ value of each SNP. Finally, SNPs with F > 10 were extracted to ensure statistical efficiency (25, 27). The F-statistic for each SNP was calculated as follows:


$$F=\left(\frac{R^{2}}{1-R^{2}}\right)*\left(\frac{n-k-1}{k}\right)$$

R^2^ was calculated as follows:


$$R^{2}=2*\left(1-MAF\right)*MAF*{\left(\beta \right)}^{2}$$

R^2^: the cumulative explained variance of the selected IVs on exposure; MAF: the effect of allele frequency.

β: estimated effect of SNP; N: sample size of the GWAS.

We utilized PhenoScanner V2 (PhenoScanner.cam.ac.uk) to eliminate SNPs that were associated with potential confounding factors of DN (27).

### Data source for diabetic nephropathy

Summary-level GWAS data pertaining to diabetic nephropathy were extracted from the Finn consortium, encompassing a cohort of 213,746 individuals of European ancestry (3,283 cases and 210,463 controls). Additionally, we gathered aggregated data on glomerular filtration rate in individuals with diabetes from a prior study, encompassing 144,935 participants of European (28). Summary statistics for urinary albumin-to-creatinine ratio with diabetes mellitus were extracted from CKDGen Consortium with 5,825 participants of European ancestry (29). To more accurately investigate the causal relationship between diabetic nephropathy and diabetic retinopathy, we acquired separate GWAS data for type 1 and type 2 diabetes respectively. Specifically, statistical summary data for type 2 diabetes with renal complications were obtained from the FinnGen database, encompassing 1,296 cases and 183,185 controls of European ancestry. For the construction of GWAS data relating to type 1 diabetes with renal disorders, summary-level GWAS data from FinnGen, comprising a total of 963 cases and 183,185 controls, were utilized.

### Statistical analysis

We employed a comprehensive range of five analytical methods, namely inverse variance weighting (IVW), MR Egger, Weighted Median (WM), Simple Mode, and Weighted Mode, to investigate the causal relationship between diabetic retinopathy and diabetic nephropathy (29). The IVW method combines the estimates from individual IVs using inverse variance weights and provides a consistent and unbiased estimation of the causal effect under the assumption that all IVs are valid and there is no violation of the instrumental variable assumptions (30). MR Egger and Weighted Median (WM) are valuable tools in Mendelian randomization studies for addressing concerns about the presence of pleiotropy or violations of instrumental variable assumptions. It allows researchers to obtain causal effect estimates while accounting for pleiotropic effects and provides additional insights into the relationship between the exposure and outcome variables. However, the Weighted Median method’s non-parametric nature may lead to less precise estimates, at the same time, MR Egger relies on regression modeling and may have reduced statistical power (31). Therefore, the result of the IVW method is the most accurate and the primary method in our study (32), while the WM and MR-Egger methods were performed as additional tests. Here we adopted the random-effects IVW model for MR estimates considering heterogeneity among instrumental variables.

Several sensitivity tests were used, including Cochran’s Q-statistic, MR Egger, MR Pleiotropy RESidual Sum, and Outlier techniques (MR-PRESSO) to obtain robust MR estimates. Cochran’s Q test was employed to assess heterogeneity among instrumental variables, with a significance level of *P*< 0.05 indicating significant heterogeneity among SNPs (33). MR-Egger analysis was employed to assess the influence of horizontal pleiotropy on the MR estimates by estimating the average pleiotropic effect and evaluating its statistical its significance (*P*< 0.05) (34). Once there is horizontal pleiotropy, MR Pleiotropy RESidual Sum and Outlier (MR-PRESSO) method will be used to identify and address outliers induced by horizontal pleiotropy. By detecting and adjusting for these outliers, MR-PRESSO enhances the precision and reliability of causal effect estimates (34). Furthermore, to evaluate the robustness and influence of individual instrumental variables on the estimated causal effect, we utilize leave-one-out cross-validation or jackknife analysis (35).

## Results

### Selection of genetic instrumental variants

We extracted 20 SNPs (single nucleotide polymorphisms) that are reliable (P< 1*10^-5^) and independent (r^2^< 0.001, kb = 10,000) of diabetic retinopathy from the FinnGen database, which included 206,234 participants of European ancestry and 16,380,446 SNPs. 5 SNPs (rs2476601, rs915894, rs3957146, rs2534659, rs689) were excluded with PhenoScannerV2 because these SNPs exhibited significant associations with established confounding factors (diabetes, HbA1c, and insulin resistance).2 SNPs (rs3957146, rs139334417) were detected as outliers for potential horizontal pleiotropy and discarded by MR-PRESSO. After Coordinating the allelic directions of Exposure-SNP and Outcome-SNP and eliminating palindromic SNP and incompatible SNP according to the size of EAF, we finally got a summary table of SNPs for background diabetic retinopathy. Refinement statistics are shown in **Supplementary Table 1**. The F-statistics of all the included SNPs were greater than 10, indicating the absence of weak instrument bias in Mendelian randomization (MR) analysis.

To further explore the causal relationship between DR and DN, summary-level GWAS data with non-proliferative background diabetic retinopathy and proliferative diabetic retinopathy were extracted.24 SNPs of non-proliferative background diabetic retinopathy were extracted as genetic instrumental variables.4 SNPs (rs6679677, rs9273401, rs9276710, rs17885785) were excluded with PhenoScannerV2 because these SNPs exhibited significant associations with established confounding factors (diabetes and insulin treatment). rs8192575 are identified as outliers by MR-PRESSO and addressed to minimize potential adverse effects on MR estimates. As for proliferative diabetic retinopathy,30 SNPs were selected as genetic instrumental variants while 7 SNPs (rs2476601, rs3957146, rs2855807, rs34337125, rs915894, rs7903146, rs689) were eliminated by PhenoScannerV2. Refinement statistics are shown in **Supplementary Tables 2.1-4.5**.

### Overall MR estimates and sensitivity analyses of DR and DN

**Figure 2:**
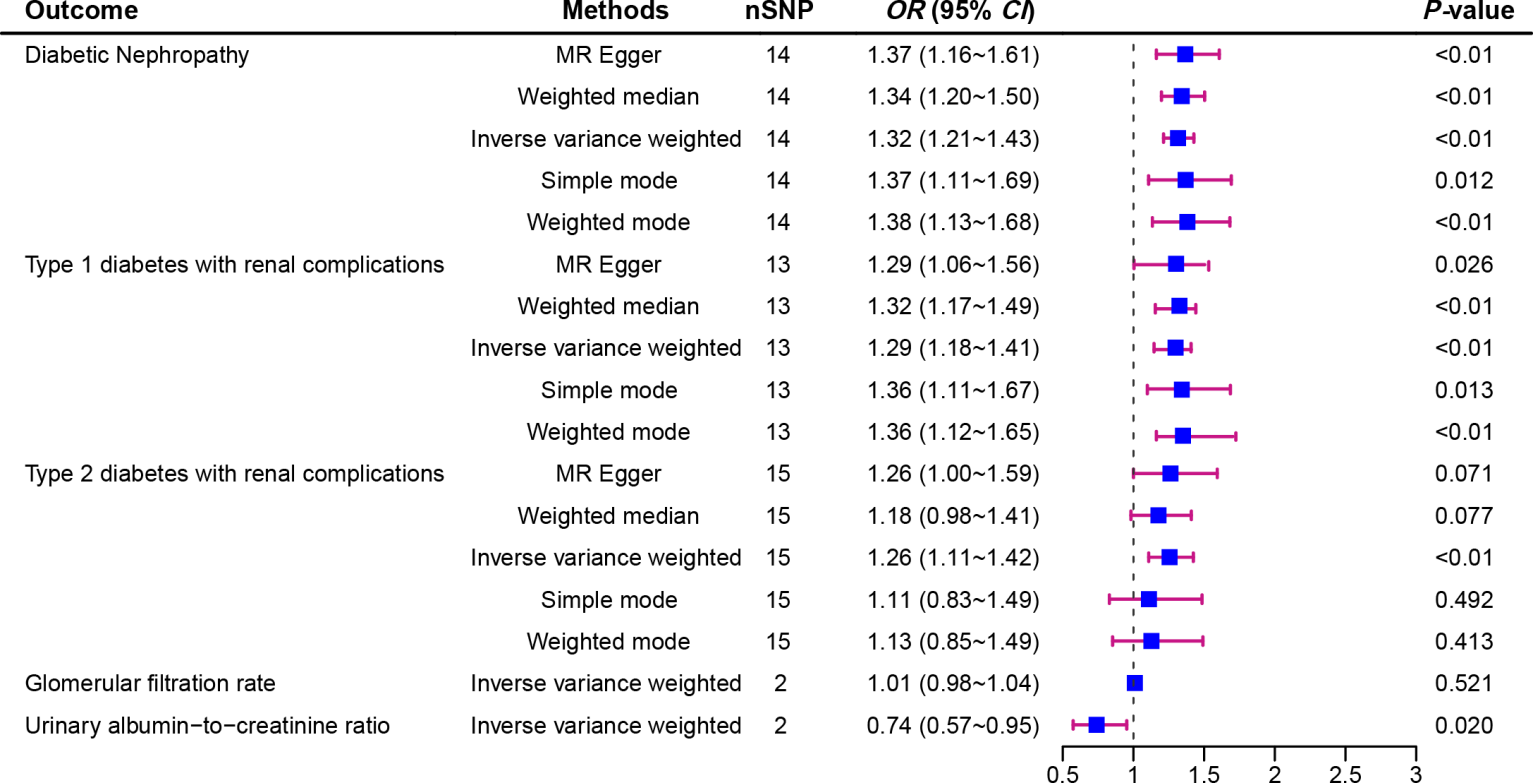
Forest plot of Mendelian randomization analyses showing the effect of diabetic retinopathy on the risk of diabetic nephropathy.

**Figure 3:**
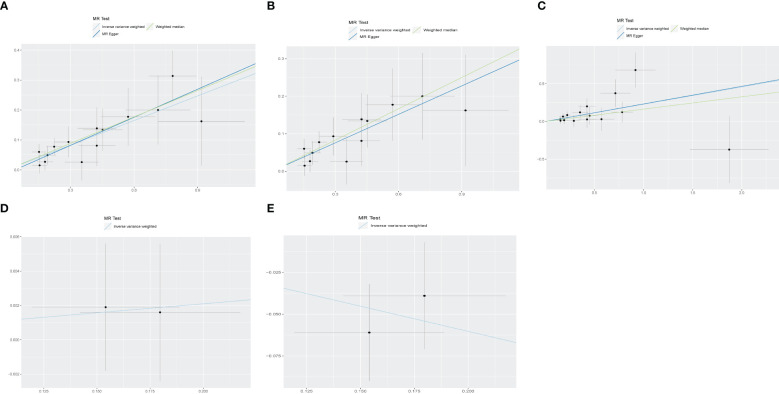
The scatter plot for MR analyses of causal associations between each diabetic retinopathy related SNP and Diabetic nephropathy **(A)**, type 1 diabetes with renal complications **(B)**, type2 diabetes with renal complications **(C)**, glomerular filtration rate in diabetics **(D)**, Glomerular filtration rate Urinary albumin−to−creatinine ratio **(E)**.

**Figure 4:**
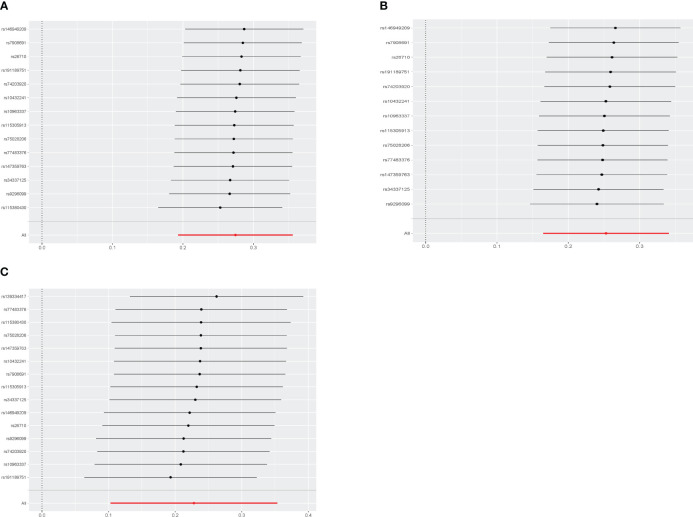
Leave-one-out sensitivity analyses of each diabetic retinopathy related SNP and Diabetic nephropathy **(A)**, type 1 diabetes with renal complications **(B)**, type2 diabetes with renal complications **(C)**.

**Figure 2** displays the estimations regarding the causal impacts of background diabetic retinopathy on diabetic nephropathy, alongside forest plots showcasing the estimations for each outcome utilizing various MR methods. The funnel plot was symmetrical, guaranteeing the robustness of the MR analysis (**Supplementary Figure 1**). Cochran’s Q statistics and MR-Egger as well as MR-PRESSO indicated no significant heterogeneity was found in our study (**Figure 3**). In the IVW method, a significant causal relationship was both found in diabetic nephropathy (OR = 1.32, 95%CI = 1.21–1.43, P = 3.72E-11), type 1 diabetes with renal complications (OR = 1.96, 95%CI = 1.60–2.41, P = 7.11E-11) and type 2 diabetes with renal complications (OR = 1.26, 95%CI = 1.11–1.42, P = 3.58E-04). The MR-Egger and WM methods reached the same conclusion in diabetic nephropathy (MR-Egger: OR=1.37, 95% CI = 1.16–1.61, P = 2.61E-03 WM: OR = 1.34,95% CI = 1.20–1.50, P = 3.67E-07) and type 1 diabetes with renal complications (MR-Egger: OR=2.01, 95% CI = 1.35–2.99, P = 4.83E-03 WM: OR = 1.70,95% CI = 1.32–2.19, P = 3.65E-05). However, no significant causal association was found in the glomerular filtration rate (OR = 1.01, 95%CI = 0.98–1.04, P = 0.521). The leave-one-out analysis further confirmed the stability of our findings, demonstrating that the outcomes were robust and not substantially influenced by any individual SNP removal (**Figure 4**).

### MR estimates and sensitivity analyses of non-proliferative DR and DN

**Figure 5:**
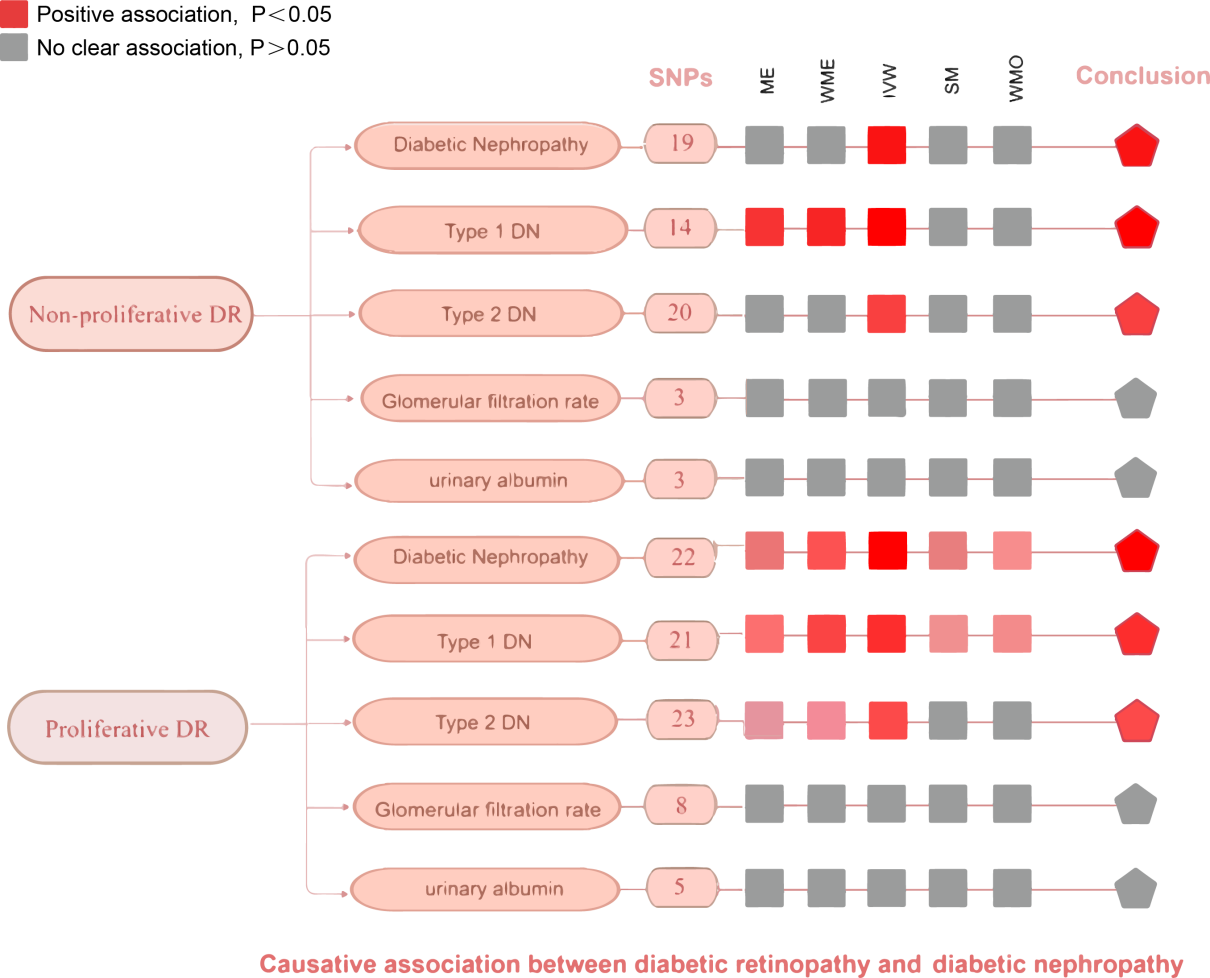
An overview of primary results from the two-sample MR studies showing the effect of non-proliferative DR and proliferative DR on the risk of diabetic nephropathy. Full data for all results depicted in the figure can be found in the **Supplementary Table S5**. IVM, inverse variance weighted method; WM, weighted median estimator; ME, MR Egger; SM, Simple mode; MO, Weighted mode.

In the study of non-proliferative DR and DN, a noteworthy causal relationship was also found in almost outcomes (**Figure 5**). The odds ratio (OR) of IVW analysis of diabetic nephropathy was 1.07(95% confidence interval [CI], 1.03~1.11; *P* = 0.000396). As for type 2 diabetes with renal complications, the IVW method also indicated a clear causality (OR = 1.07, 95%CI = 1.01–1.14, P = 0.02285986). The result of IVW (OR= 5.257, *P*= 0.017), MR-Egger (OR= 5.257, *P*= 0.017) and Weight-Median (OR=2.818, 95% CI:1.145-6.936, *P*= 0.024) reached the same conclusion, indicating a significant positive causal connection were identified in coffee intake and diabetic nephropathy. However, no significant causal association was found in glomerular filtration rate (OR = 0.99, 95%CI = 0.98–1.01, P = 0.323) and urinary albumin (OR = 1.04, 95%CI = 0.91–1.19, P = 0.589). No indication of heterogeneity and horizontal pleiotropy was found in all five outcomes (**Supplementary Figures 3, 4**). These finding was all robust in the leave-one-out sensitivity analysis (**Supplementary Figure 2**).

### MR estimates and sensitivity analyses of proliferative DR and DN

We conducted the same analysis for the database about proliferative DR and DN and reached a similar conclusion eventually (**Figure 5**). The IVW method indicates a significant causal relationship both in diabetic nephropathy (OR = 1.67, 95%CI = 1.46–1.90, P = 3.699068E-14), type 1 diabetes with renal complications (OR = 2.99, 95%CI = 2.23–4.00, P=1.945534E-13), type 2 diabetes with renal complications (OR = 1.83, 95%CI = 1.46–2.29, P = 1.807069E-07). Similar to the results of IVW analyses, the MR-Egger (OR=1.90, 95% CI: 1.38-2.63, *P*=8.623717E-04) and WM analyses (OR=1.70, 95% CI: 1.39-2.09, *P*=2.967800E-07) suggested a relationship of a distinctive character between proliferative DR and diabetic nephropathy. When it comes to type 1/2 diabetes with renal complications, the MR-Egger and WM analyses also yielded identical positive outcomes. The *P* value of Cochran’s Q test was 0.5113727, 0.1362339, and 0.2482583 respectively, indicating that the heterogeneity was low and insignificant. The pleiotropy analysis using the MR-Egger intercept test and MR-PRESSO showed that all genetic variants used had no substantial pleiotrophin in diabetic nephropathy (Egger intercept = -0.01609629, *P* = 0.3885723; global test *P*=0.6417), type 1 diabetes with renal complications y (Egger intercept =-0.06371645, *P* =0.1453258; global test *P*=0.23) and type 2 diabetes with renal complications (Egger intercept =-0.005553915, *P* = 0.8617735; global test *P*= 0.3243333). The sensitivity analysis with the leave-one-out approach found that the pooled effect estimates changed insignificantly, ensuring the robustness and reliability of the outcomes (**Supplementary Figure 5**). However, random effects IVW estimates indicated that more severity of proliferative DR is not causally associated with a higher risk of glomerular filtration rate in diabetics (OR= 1.00, 95% CI: 0. 96-1.04, *P*=0.960) or urinary albumin (OR= 1.12, 95% CI: 0.77-1.62 *P*=0.553). Sensitivity tests suggest neither heterogeneity nor polymorphism in our MR analysis.

### Multivariable MR analysis

Considering DR and DN are microvascular complications of diabetes caused by chronic hyperglycaemia, we embarked on a comprehensive investigation by incorporating two prevalent risk factors, fasting glucose and HbA1c, into our MVMR analysis. The utilization of the MVMR methodology serves the purpose of elucidating the intricate interplay among these risk factors, thus enhancing the robustness and credibility of our study. Within the framework of the MVMR analysis, while meticulously controlling for HbA1c, a direct causal link between background diabetic retinopathy and the heightened susceptibility to both DN (IVW OR = 1.57, 95% CI = 1.47-1.68, P = 2.98E-43) and Type 2 diabetes accompanied by renal complications (IVW OR = 1.22, 95% CI = 1.11-1.34, P = 3.87E-05) was affirmed. Furthermore, subsequent adjustments for both HbA1c and fasting glucose unveiled substantial support for a direct causal effect of proliferative DR on the risk of DN (HbA1c: IVW OR = 2.13, 95% CI= 1.96-2.31, P< 0.001; fasting glucose: IVW OR = 2.16, 95% CI= 1.70-2.73, P< 0.001) (**Supplementary Table 8**).

## Discussion

The present study demonstrated a significant association between genetically predicted diabetic retinopathy and an increased risk of diabetic nephropathy. Furthermore, the results of two MR study confirmed that there was indeed a causal association both in non-proliferative DR and proliferative DR. The observed correlations between DR and DN encompass shared pathogenic mechanisms, pathological outcomes, and clinical manifestations. The utilization of artificial intelligence and machine learning in quantifying retinal vascular geometric parameters holds promise in providing new insights for the diagnosis and differentiation of DN. Our study provides a genetic basis for the diagnosis of diabetic nephropathy in diabetic retinopathy.

This finding was in line with the outcomes of a prospective population-based study in 2020, in which bilateral fundic photographs of 91 Chinese type 2 diabetic patients with biopsy-confirmed DN were obtained to estimate the hazard ratio for the effect of the severity of diabetic retinopathy on ESRD. The experiment results show that the severity of retinopathy at the time of biopsy was a prognostic factor for progression to ESRD (HR 2.18, 95% confidence interval 1.05 to 4.53, P = 0.04) (15). Likewise, a systematic review by Pearce et al. demonstrated a strong association between DR and DN. The presence of DR increases the risk of developing nephropathy and serves as a predictive indicator for the progression of both microalbuminuria and macroalbuminuria (36). Another meta-analysis revealed that the sensitivity of DR in distinguishing between DN nondiabeticetic renal disease (NDRD) in type 2 diabetes patients is 0.65, with a specificity of 0.75. Importantly, it emphasized the high specificity of DR diagnosis for PDR in identifying DN (37). Yamanouchi et al. observed that with increasing severity of DR (from no DR, non-proliferative DR, to proliferative DR), the grading of DN based on renal pathological changes such as glomerular injury, interstitial fibrosis and tubular atrophy (IFTA) scores, as well as diffuse lesion scores, progressively increased. This suggests that the severity of DR could serve as a clinical indicator for predicting the severity of renal pathology (38). However, in another study, the correlation between DR and Grade IIb and III glomerular lesions was significantly higher than that with Grade IV lesions, possibly due to differences in the mechanisms of glomerular nodular sclerosis and global glomerulosclerosis. The specific reasons require further investigation (39). In retinal fundus photographs, calculations of the central artery and retinal central vein dimensions, as well as the arteriovenous ratio (AVR), can provide indications of cardiovascular risk (40). Similarly, several studies have attempted to predict renal pathological changes through fundus examination. Multiple cross-sectional studies have demonstrated an association between the enlargement of retinal vein diameter and the severity of both DR and DN (41). Erdogmus et al. reported that DR has a sensitivity of 75%, specificity of 91%, positive predictive value of 88%, and negative predictive value of 81% in predicting DN. Multivariable regression analysis suggested that DR is an independent predictor of DN (42). However, other studies have demonstrated inconsistencies between DN and DR manifestations. Christensen et al. reported that albuminuria patients with type 2 diabetes without DR have a prevalence of biopsies with normal glomerular structure or nondiabetic kidney diseases of approximately 30% (43). These contradictory findings observed may be attributed to inherent biases or confounders intrinsic in observational studies, including limitations stemming from small sample sizes, heterogeneity in demographic characteristics, variations in study designs, reverse causation, and selection bias, such as differences in methods used to assess DR. It is important to note that observational studies primarily analyze correlations rather than establishing causation. To mitigate the impact of potential bias and confounders, MR analysis was conducted to investigate the causal association between DR and DN from a genetic standpoint. In our study, there was remarkable evidence supporting the fact that DR was causally associated with DN.

Several potential pathways have been proposed to elucidate the mechanisms underlying the causality between DR and DN. Advanced glycation end products (AGEs), a heterogeneous group of molecules that form through a series of non-enzymatic reactions between reducing sugars and proteins, lipids, or nucleic acids, once bound to the receptor for AGEs (RAGE), can initiate multiple signaling pathways that contribute to microvascular damage. The AGE-RAGE complex can activate signaling pathways such as reactive oxygen species (ROS) and mitogen-activated protein kinase (MAPK) in various cells. This activation leads to increased inflammation and proliferation of mesangial cells in the kidneys, ultimately resulting in apoptosis (44). In DR, the AGE-RAGE complex induces oxidative stress, osteogenic differentiation, calcification in pericytes, and also triggers apoptosis (45). On top of the AGE-RAGE component with excess glucose in the cell, DAG is formed, which subsequently triggers the activation of protein kinase C (PKC) (46). Lin et al. demonstrated that activated protein kinase C (aPKC) can induce the release of endothelin and VEGF, leading to increased vascular permeability in animal models of retinal inflammation (47). Wang et al. also mentioned LY333531, a PKCβ inhibitor, can promote the degradation of type IV collagen and fibronectin while downregulate the expression of the pro-apoptotic protein swiprosin-1, attenuating glomerular endothelial cell apoptosis (48). Additionally, SHP-1 is a class of protein tyrosine phosphatases with an SH2 domain that controls intracellular phosphorylation levels of tyrosine (49). High glucose levels can activate PKCδ/p38α MAPK, leading to independent activation of downstream NFκB and activation of SHP-1, ultimately resulting in the development of DR (50). Likewise, elevated glucose levels were also reported to activate SHP-1 activity and inhibit VEGF in podocytes, thereby contributing to glomerular disease progression in diabetes (51).

Nowadays, an increasing body of clinical and fundamental research has accumulated compelling evidence supporting a significant correlation between DN and DR. To the best of our knowledge, this study represents the first large-scale Mendelian randomization investigation focusing on the relationship between DR and DN. Our study possesses several distinct advantages. Firstly, the utilization of MR methodology, considered as a natural randomized controlled trial approach, provides more robust evidence compared to previous observational studies. Secondly, our study exclusively focuses on individuals of European descent, thereby minimizing potential biases arising from population stratification. Moreover, we conducted a staging study of disease progression aiming to explore the causal relationship between DR and DN precisely. A comprehensive set of sensitivity analyses, including MR Egger, Cochran’s Q test, MR-PRESSO, and leave-one-out analysis, consistently demonstrated the robustness of the findings.

This study has several limitations that should be acknowledged. Firstly, despite utilizing the largest available sample size and the most recent GWAS dataset for MR analysis, it is important to recognize that our study had a relatively smaller sample size and event count when compared to population-based observational studies. Secondly, meeting three assumptions is crucial. Of particular difficulty is fulfilling the second assumption, which necessitates that instrumental variables should not be associated with confounding factors. To address this concern, we employed PhenoScanner to manually exclude SNPs associated with known confounders, thereby striving to ensure reliable and plausible results. However, it is impractical to account for all potential confounding factors exhaustively. Thirdly, Systems biology contains complex interactions between genes, proteins, and other molecules, as well as the influence of environmental factors, thus incorporating data on developmental compensation and epigenetic phenomena would provide a more comprehensive and accurate understanding of causality.

## Conclusion

In conclusion, our MR study confirms a significant correlation between DR and DN based on genetic evidence, highlighting the non-invasive nature and relatively accurate diagnostic capability of diabetic retinopathy in predicting diabetic nephropathy. Future research efforts should involve long-term extensive multicenter cohort studies to validate biomarkers and mechanistic studies to confirm the association between DR and DN in terms of severity and disease progression, which will provide more effective assistance in the early non-invasive detection and real-time monitoring of DN and in slowing down its progression.

## Data availability statement

The original contributions presented in the study are included in the article/**Supplementary Material**. Further inquiries can be directed to the corresponding authors.

## Ethics statement

Ethical approval was not required for the study involving humans in accordance with the local legislation and institutional requirements. Written informed consent to participate in this study was not required from the participants or the participants’ legal guardians/next of kin in accordance with the national legislation and the institutional requirements.

## Author contributions

JF: Writing – original draft. CL: Writing – original draft. DZ: Writing – original draft. QH: Writing – review & editing. LL: Writing – review & editing.
