# Supplementary material for: Correlation between diabetic retinopathy and diabetic nephropathy: a two-sample Mendelian randomization study
Source: Front Endocrinol (Lausanne). 2023 Nov 1;14:1265711. doi: 10.3389/fendo.2023.1265711 (PMC10646564; doi:10.3389/fendo.2023.1265711)
Supplement: Supplementary file 1 [file Image_1.pdf]

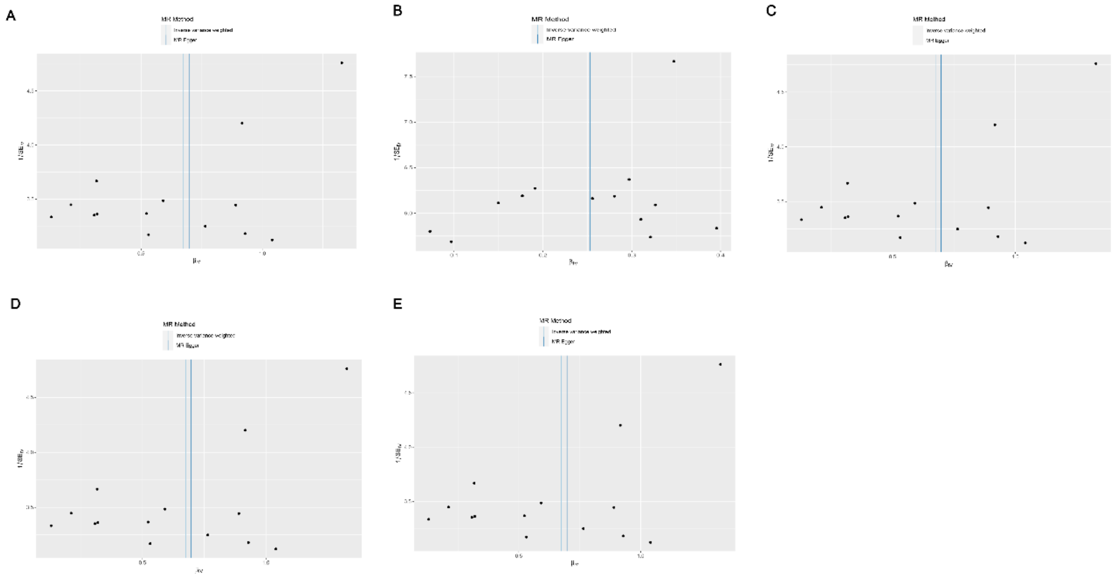

Supplementary Figure 1. Funnel plots of diabetic retinopathy on the risk of diabetic nephropathy. (A) Diabetic nephropathy, (B)type 1 diabetes with renal complications, (C) type2 diabetes with renal complications, (D) glomerular filtration rate in diabetics, (E)Glomerular filtration rate Urinary albumin−to−creatinine ratio. The funnel plots exhibit symmetry, indicating the absence of polymorphism

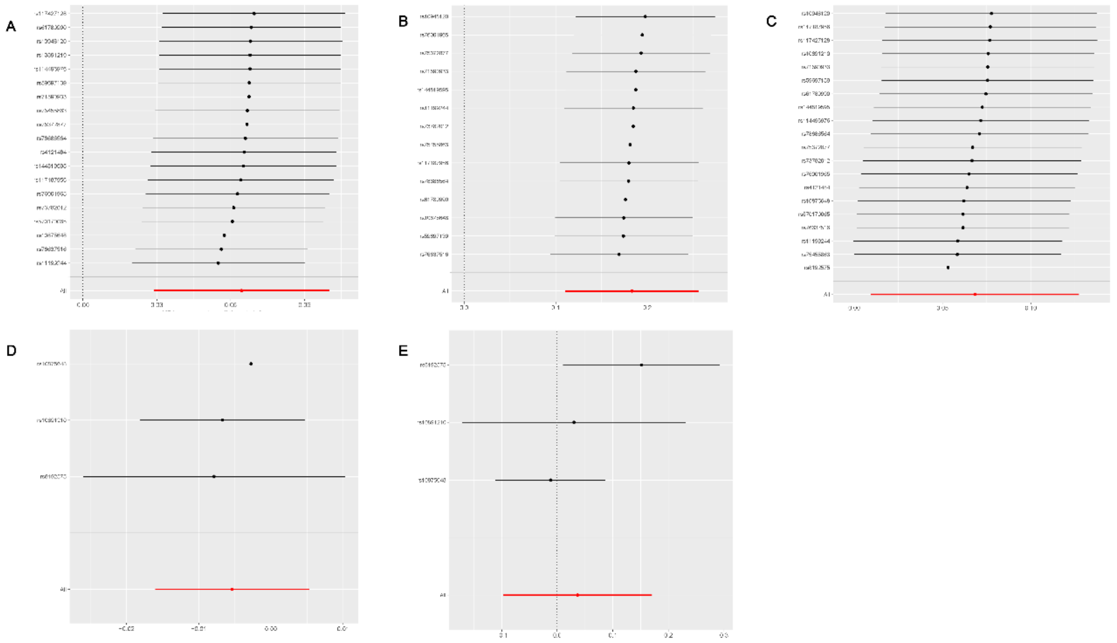

Supplementary Figure 2. Leave-one-out sensitivity analyses of each non-proliferative DR related SNP and Diabetic nephropathy(A), type 1 diabetes with renal complications(B), type2 diabetes with renal complications(C), glomerular filtration rate in diabetics (D), Glomerular filtration rate Urinary albumin−to−creatinine ratio (E).

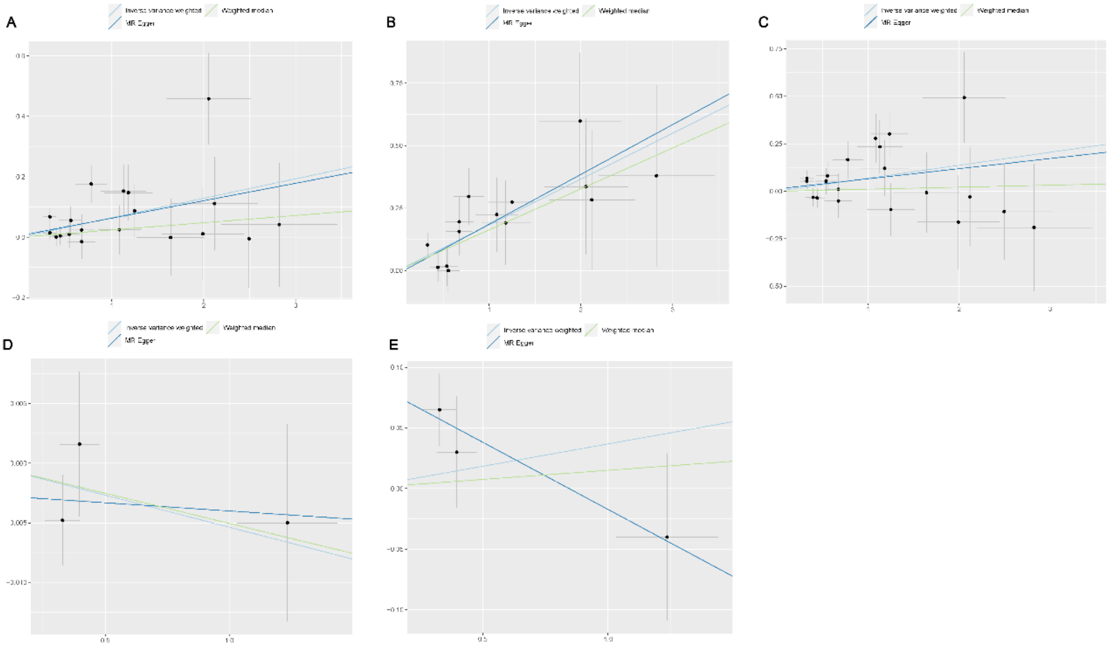

Supplementary Figure 3 The scatter plot for MR analyses of causal associations between each non-proliferative DR related SNP and Diabetic nephropathy(A), type 1 diabetes with renal complications(B), type2 diabetes with renal complications(C), glomerular filtration rate in diabetics (D), Glomerular filtration rate Urinary albumin−to−creatinine ratio (E).

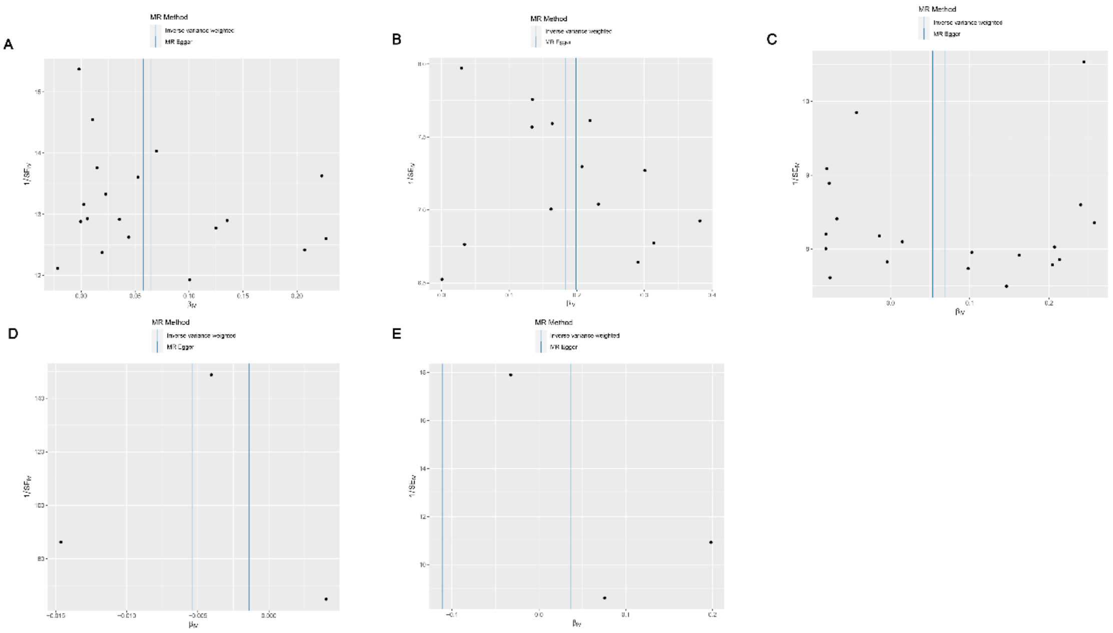

Supplementary Figure 4. Funnel plots of diabetic retinopathy on the risk of non-proliferative DR. (A) Diabetic nephropathy, (B)type 1 diabetes with renal complications, (C) type2 diabetes with renal complications, (D) glomerular filtration rate in diabetics, (E)Glomerular filtration rate Urinary albumin−to−creatinine ratio.

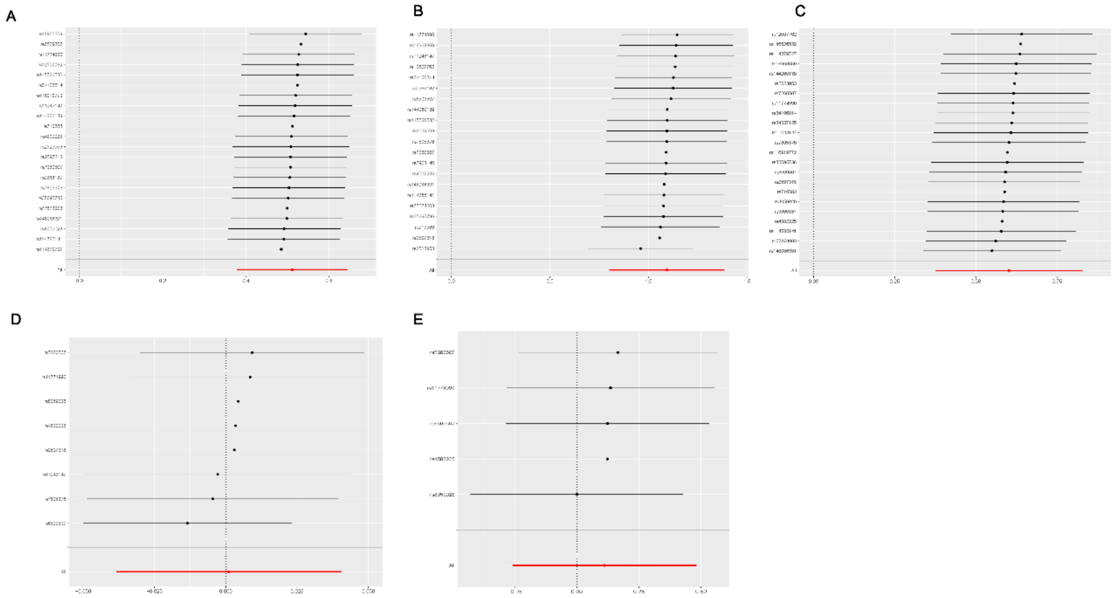

Supplementary Figure 5. Leave-one-out sensitivity analyses of each proliferative DR related SNP and Diabetic nephropathy(A), type 1 diabetes with renal complications(B), type2 diabetes with renal complications(C), glomerular filtration rate in diabetics (D), Glomerular filtration rate Urinary albumin−to−creatinine ratio (E).

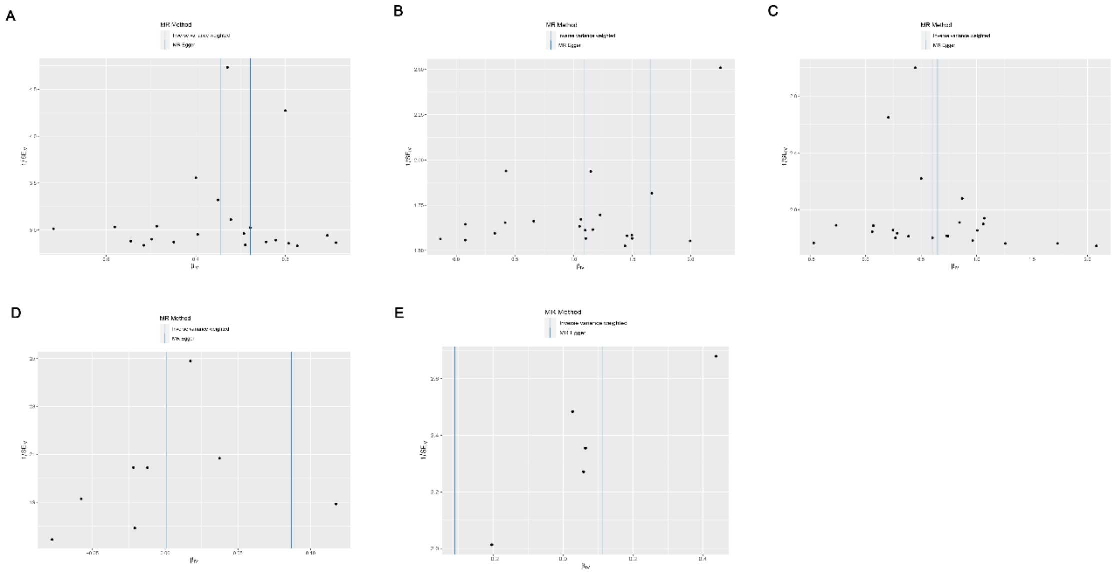

Supplementary Figure 6. Funnel plots of diabetic retinopathy on the risk of proliferative DR. (A) Diabetic nephropathy, (B)type 1 diabetes with renal complications, (C) type2 diabetes with renal complications, (D) glomerular filtration rate in diabetics, (E)Glomerular filtration rate Urinary albumin−to−creatinine ratio.

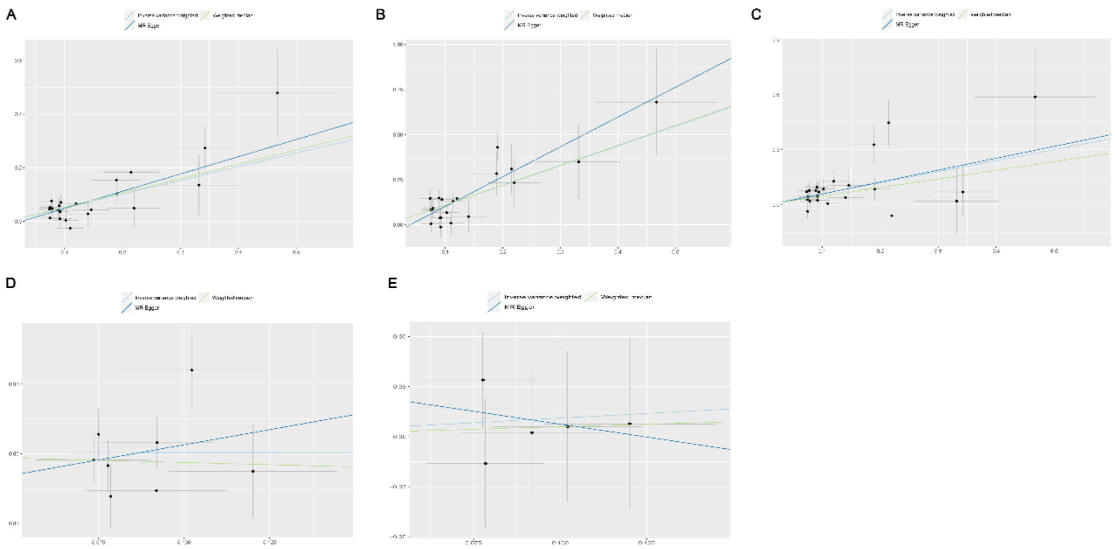

Supplementary Figure 7 The scatter plot for MR analyses of causal associations between each proliferative DR related SNP and Diabetic nephropathy(A), type 1 diabetes with renal complications(B), type2 diabetes with renal complications(C), glomerular filtration rate in diabetics (D), Glomerular filtration rate Urinary albumin−to−creatinine ratio (E).
