# Supplementary material for: Correlation between diabetic retinopathy and diabetic nephropathy: a two-sample Mendelian randomization study
Source: Front Endocrinol (Lausanne). 2023 Nov 1;14:1265711. doi: 10.3389/fendo.2023.1265711 (PMC10646564; doi:10.3389/fendo.2023.1265711)
Supplement: Supplementary file 2 [file Table_1.docx]

**Table S1** Characteristics of instrumental variables for Background diabetic retinopathy

|  | **SNP** | **EA** | **OA** | **Samplesize** | **SE** | **β** | **EAF** | ***P* value** | **R^2^** | **F - statistic** |
| --- | --- | --- | --- | --- | --- | --- | --- | --- | --- | --- |
| 1 | rs77483376 | G | A | 206,234 | 0.0655 | 0.2903 | 0.07012 | 9.23E-06 | 0.010989878 | 2291.671747 |
| 2 | rs139334417 | T | C | 206,234 | 0.3994 | 1.8728 | 0.002341 | 2.74E-06 | 0.01638311 | 3435.030701 |
| 3 | rs26710 | T | C | 206,234 | 0.0377 | -0.1797 | 0.2536 | 1.93E-06 | 0.012224948 | 2552.402974 |
| 4 | rs115305913 | T | G | 206,234 | 0.0931 | 0.4516 | 0.03407 | 1.23E-06 | 0.013423187 | 2805.982845 |
| 5 | rs115380430 | C | A | 206,234 | 0.1111 | 0.7822 | 0.0234 | 1.90E-12 | 0.027963929 | 5933.02366 |
| 6 | rs9296099 | A | G | 206,234 | 0.0372 | 0.2231 | 0.2646 | 1.97E-09 | 0.019370579 | 4073.783529 |
| 7 | rs147359763 | C | T | 206,234 | 0.0912 | 0.4245 | 0.03571 | 3.23E-06 | 0.012410318 | 2591.591931 |
| 8 | rs10963337 | A | G | 206,234 | 0.1533 | 0.7132 | 0.01265 | 3.30E-06 | 0.01270616 | 2654.166471 |
| 9 | rs7908691 | G | T | 206,234 | 0.0348 | 0.1541 | 0.6675 | 9.50E-06 | 0.010540912 | 2197.053418 |
| 10 | rs191189751 | C | T | 206,234 | 0.2047 | 0.9179 | 0.007549 | 7.31E-06 | 0.012624647 | 2636.921598 |
| 11 | rs75028206 | T | C | 206,234 | 0.1276 | 0.5718 | 0.01772 | 7.38E-06 | 0.011381967 | 2374.373595 |
| 12 | rs34337125 | A | G | 206,234 | 0.0334 | -0.1517 | 0.4147 | 5.60E-06 | 0.011171557 | 2329.9845 |
| 13 | rs146949209 | T | C | 206,234 | 0.0793 | -0.3532 | 0.04625 | 8.51E-06 | 0.0110057 | 2295.007717 |
| 14 | rs10432241 | A | G | 206,234 | 0.04 | -0.1916 | 0.7813 | 1.68E-06 | 0.01254549 | 2620.177903 |
| 15 | rs74203920 | T | C | 206,234 | 0.0875 | 0.424 | 0.03722 | 1.26E-06 | 0.012884428 | 2691.890561 |

SNP:single nucleotide polymorphisms; EA: effect allele; OA: other allele; EAF: effect allele frequency; SE, standard error

**Table S2.1** SNPs from GWAS on Background diabetic retinopathy and Diabetic nephropathy

|  | | | **Exposure (Background diabetic retinopathy)** | | |  | **Outcome (Diabetic nephropathy)** | | | | |
| --- | --- | --- | --- | --- | --- | --- | --- | --- | --- | --- | --- |
| **SNP** | **EA** | **OA** | **β** | **SE** | ***P* value** |  | **Case** | **Control** | **β** | **SE** | ***P* value** |
| rs10432241 | A | G | -0.1916 | 0.04 | 1.68E-06 |  | 3,283 | 210,463 | -0.049 | 0.0311 | 0.1154 |
| rs10963337 | A | G | 0.7132 | 0.1533 | 3.30E-06 |  | 3,283 | 210,463 | 0.2 | 0.1153 | 0.082781 |
| rs115305913 | T | G | 0.4516 | 0.0931 | 1.23E-06 |  | 3,283 | 210,463 | 0.1341 | 0.0709 | 0.058721 |
| rs115380430 | C | A | 0.7822 | 0.1111 | 1.90E-12 |  | 3,283 | 210,463 | 0.3137 | 0.085 | 0.000224 |
| rs146949209 | T | C | -0.3532 | 0.0793 | 8.51E-06 |  | 3,283 | 210,463 | -0.0257 | 0.0609 | 0.672901 |
| rs147359763 | C | T | 0.4245 | 0.0912 | 3.23E-06 |  | 3,283 | 210,463 | 0.1386 | 0.0697 | 0.04659 |
| rs191189751 | C | T | 0.9179 | 0.2047 | 7.31E-06 |  | 3,283 | 210,463 | 0.1622 | 0.1483 | 0.2742 |
| rs26710 | T | C | -0.1797 | 0.0377 | 1.93E-06 |  | 3,283 | 210,463 | -0.0269 | 0.0294 | 0.3601 |
| rs34337125 | A | G | -0.1517 | 0.0334 | 5.60E-06 |  | 3,283 | 210,463 | -0.06 | 0.026 | 0.02114 |
| rs74203920 | T | C | 0.424 | 0.0875 | 1.26E-06 |  | 3,283 | 210,463 | 0.081 | 0.0676 | 0.2309 |
| rs75028206 | T | C | 0.5718 | 0.1276 | 7.38E-06 |  | 3,283 | 210,463 | 0.1774 | 0.0964 | 0.06582 |
| rs77483376 | G | A | 0.2903 | 0.0655 | 9.23E-06 |  | 3,283 | 210,463 | 0.0932 | 0.0506 | 0.065489 |
| rs7908691 | G | T | 0.1541 | 0.0348 | 9.50E-06 |  | 3,283 | 210,463 | 0.0149 | 0.0271 | 0.5819 |
| rs9296099 | A | G | 0.2231 | 0.0372 | 1.97E-09 |  | 3,283 | 210,463 | 0.0775 | 0.0291 | 0.007702 |

SNP:single nucleotide polymorphisms; EA: effect allele; OA: other allele; SE, standard error

**Table S2.2** SNPs from GWAS on Background diabetic retinopathy and Type 1 diabetes with renal complications

|  | | | **Exposure (Background diabetic retinopathy)** | | |  | **Outcome (Type 1 diabetes with renal complications)** | | | | |
| --- | --- | --- | --- | --- | --- | --- | --- | --- | --- | --- | --- |
| **SNP** | **EA** | **OA** | **β** | **SE** | ***P* value** |  | **Case** | **Control** | **β** | **SE** | ***P* value** |
| rs10432241 | A | G | -0.1916 | 0.04 | 1.68E-06 |  | 963 | 183,185 | -0.0611 | 0.057 | 0.2835 |
| rs10963337 | A | G | 0.7132 | 0.1533 | 3.30E-06 |  | 963 | 183,185 | 0.219 | 0.2128 | 0.3035 |
| rs115305913 | T | G | 0.4516 | 0.0931 | 1.23E-06 |  | 963 | 183,185 | 0.267 | 0.1296 | 0.0393396 |
| rs139334417 | T | C | 1.8728 | 0.3994 | 2.74E-06 |  | 963 | 183,185 | 0.5926 | 0.5107 | 0.2459 |
| rs146949209 | T | C | -0.3532 | 0.0793 | 8.51E-06 |  | 963 | 183,185 | -0.1876 | 0.1114 | 0.0922699 |
| rs147359763 | C | T | 0.4245 | 0.0912 | 3.23E-06 |  | 963 | 183,185 | 0.2218 | 0.1261 | 0.0785796 |
| rs191189751 | C | T | 0.9179 | 0.2047 | 7.31E-06 |  | 963 | 183,185 | 0.1936 | 0.2663 | 0.4673 |
| rs26710 | T | C | -0.1797 | 0.0377 | 1.93E-06 |  | 963 | 183,185 | -0.0233 | 0.0539 | 0.665301 |
| rs34337125 | A | G | -0.1517 | 0.0334 | 5.60E-06 |  | 963 | 183,185 | -0.141 | 0.0477 | 0.00310899 |
| rs74203920 | T | C | 0.424 | 0.0875 | 1.26E-06 |  | 963 | 183,185 | 0.3776 | 0.1231 | 0.002157 |
| rs75028206 | T | C | 0.5718 | 0.1276 | 7.38E-06 |  | 963 | 183,185 | 0.4373 | 0.176 | 0.01295 |
| rs77483376 | G | A | 0.2903 | 0.0655 | 9.23E-06 |  | 963 | 183,185 | 0.3021 | 0.093 | 0.00116099 |
| rs9296099 | A | G | 0.2231 | 0.0372 | 1.97E-09 |  | 963 | 183,185 | 0.2045 | 0.0531 | 0.00011650 |

SNP:single nucleotide polymorphisms; EA: effect allele; OA: other allele; SE, standard error

**Table S2.3** SNPs from GWAS on Background diabetic retinopathy and Type2 diabetes with renal complications

|  | | | **Exposure (Background diabetic retinopathy)** | | |  | **Outcome (Type2 diabetes with renal complications)** | | | | |
| --- | --- | --- | --- | --- | --- | --- | --- | --- | --- | --- | --- |
| **SNP** | **EA** | **OA** | **β** | **SE** | ***P* value** |  | **Case** | **Control** | **β** | **SE** | ***P* value** |
| rs10432241 | A | G | -0.1916 | 0.04 | 1.68E-06 |  | 1,296 | 183,185 | -0.0159 | 0.0499 | 0.749899 |
| rs10963337 | A | G | 0.7132 | 0.1533 | 3.30E-06 |  | 1,296 | 183,185 | 0.3738 | 0.1825 | 0.0406 |
| rs115305913 | T | G | 0.4516 | 0.0931 | 1.23E-06 |  | 1,296 | 183,185 | 0.0756 | 0.114 | 0.507399 |
| rs115380430 | C | A | 0.7822 | 0.1111 | 1.90E-12 |  | 1,296 | 183,185 | 0.123 | 0.1378 | 0.3719 |
| rs139334417 | T | C | 1.8728 | 0.3994 | 2.74E-06 |  | 1,296 | 183,185 | -0.3729 | 0.4408 | 0.3977 |
| rs146949209 | T | C | -0.3532 | 0.0793 | 8.51E-06 |  | 1,296 | 183,185 | -0.1201 | 0.0972 | 0.217 |
| rs147359763 | C | T | 0.4245 | 0.0912 | 3.23E-06 |  | 1,296 | 183,185 | 0.0267 | 0.1115 | 0.811 |
| rs191189751 | C | T | 0.9179 | 0.2047 | 7.31E-06 |  | 1,296 | 183,185 | 0.6807 | 0.2322 | 0.003378 |
| rs26710 | T | C | -0.1797 | 0.0377 | 1.93E-06 |  | 1,296 | 183,185 | -0.0645 | 0.0468 | 0.1684 |
| rs34337125 | A | G | -0.1517 | 0.0334 | 5.60E-06 |  | 1,296 | 183,185 | -0.029 | 0.0414 | 0.4831 |
| rs74203920 | T | C | 0.424 | 0.0875 | 1.26E-06 |  | 1,296 | 183,185 | 0.1995 | 0.1098 | 0.0693 |
| rs75028206 | T | C | 0.5718 | 0.1276 | 7.38E-06 |  | 1,296 | 183,185 | 0.0296 | 0.1543 | 0.8478 |
| rs77483376 | G | A | 0.2903 | 0.0655 | 9.23E-06 |  | 1,296 | 183,185 | 0.0082 | 0.0809 | 0.9189 |
| rs7908691 | G | T | 0.1541 | 0.0348 | 9.50E-06 |  | 1,296 | 183,185 | 0.0108 | 0.0432 | 0.8029 |
| rs9296099 | A | G | 0.2231 | 0.0372 | 1.97E-09 |  | 1,296 | 183,185 | 0.0841 | 0.0465 | 0.07046 |

SNP:single nucleotide polymorphisms; EA: effect allele; OA: other allele; SE, standard error

**Table S2.4** SNPs from GWAS on Background diabetic retinopathy and Glomerular filtration rate in diabetics

|  | | | **Exposure (Background diabetic retinopathy)** | | |  | **Outcome (Glomerular filtration rate in diabetics)** | | | | |
| --- | --- | --- | --- | --- | --- | --- | --- | --- | --- | --- | --- |
| **SNP** | **EA** | **OA** | **β** | **SE** | ***P* value** |  | **Case** | **Control** | **β** | **SE** | ***P* value** |
| rs26710 | T | C | -0.1797 | 0.0377 | 1.93E-06 |  | 11,522 | 133,413 | -0.0016 | 0.004 | 0.68 |
| rs7908691 | G | T | 0.1541 | 0.0348 | 9.50E-06 |  | 11,522 | 133,413 | 0.0019 | 0.0037 | 0.61 |

SNP:single nucleotide polymorphisms; EA: effect allele; OA: other allele; SE, standard error

**Table S2.5** SNPs from GWAS on Background diabetic retinopathy and albumin-to-creatinine ratio in diabetics

|  | | | **Exposure (Background diabetic retinopathy)** | | |  | **Outcome (albumin-to-creatinine ratio in diabetics)** | | | | |
| --- | --- | --- | --- | --- | --- | --- | --- | --- | --- | --- | --- |
| **SNP** | **EA** | **OA** | **β** | **SE** | ***P* value** |  | **Case** | **Control** | **β** | **SE** | ***P* value** |
| rs26710 | T | C | -0.1797 | 0.0377 | 1.93E-06 |  | 5,825 | 46061 | 0.039 | 0.032 | 0.22 |
| rs7908691 | G | T | 0.1541 | 0.0348 | 9.50E-06 |  | 5,825 | 46061 | -0.061 | 0.029 | 0.038 |

SNP:single nucleotide polymorphisms; EA: effect allele; OA: other allele; SE, standard error

**Table S3.1 SNPs from GWAS on non-proliferative background diabetic retinopathy and Diabetic nephropathy**

|  | | | **Exposure (non-proliferative DR)** | | |  | **Outcome (Diabetic nephropathy)** | | | | |
| --- | --- | --- | --- | --- | --- | --- | --- | --- | --- | --- | --- |
| **SNP** | **EA** | **OA** | **β** | **SE** | ***P* value** |  | **Case** | **Control** | **β** | **SE** | ***P* value** |
| rs10875648 | A | G | 0.3278 | 0.0699 | 2.72E-06 |  | 3,283 | 210,463 | 0.0679 | 0.0264 | 0.01017 |
| rs10891210 | T | G | -0.3962 | 0.0796 | 6.52E-07 |  | 3,283 | 210,463 | -9.00E-04 | 0.0301 | 0.9764 |
| rs10948120 | A | G | 0.4408 | 0.0812 | 5.74E-08 |  | 3,283 | 210,463 | 0.0046 | 0.0303 | 0.8786 |
| rs11199244 | A | G | 2.0551 | 0.4567 | 6.80E-06 |  | 3,283 | 210,463 | 0.4584 | 0.1508 | 0.002371 |
| rs114496976 | G | T | 1.6411 | 0.3698 | 9.08E-06 |  | 3,283 | 210,463 | -9.00E-04 | 0.1274 | 0.9941 |
| rs117187956 | A | T | 1.2491 | 0.2605 | 1.63E-06 |  | 3,283 | 210,463 | 0.0873 | 0.089 | 0.3271 |
| rs117427128 | G | T | 2.4939 | 0.5307 | 2.61E-06 |  | 3,283 | 210,463 | -0.005 | 0.1622 | 0.9752 |
| rs144819595 | T | G | 2.1175 | 0.4703 | 6.72E-06 |  | 3,283 | 210,463 | 0.1114 | 0.1556 | 0.4738 |
| rs4121484 | T | G | 0.327 | 0.0685 | 1.79E-06 |  | 3,283 | 210,463 | 0.0144 | 0.0259 | 0.5782 |
| rs570170065 | C | T | 1.1285 | 0.2463 | 4.60E-06 |  | 3,283 | 210,463 | 0.1527 | 0.0875 | 0.081171 |
| rs59697139 | G | A | 1.9907 | 0.4487 | 9.14E-06 |  | 3,283 | 210,463 | 0.0114 | 0.154 | 0.9409 |
| rs61780990 | T | C | 0.6735 | 0.1522 | 9.60E-06 |  | 3,283 | 210,463 | -0.0147 | 0.0556 | 0.7914 |
| rs71590933 | A | T | 2.8221 | 0.6321 | 8.01E-06 |  | 3,283 | 210,463 | 0.0418 | 0.2051 | 0.8384 |
| rs73782012 | C | T | 1.1805 | 0.265 | 8.40E-06 |  | 3,283 | 210,463 | 0.1476 | 0.0924 | 0.1104 |
| rs75372877 | T | A | 0.5384 | 0.116 | 3.48E-06 |  | 3,283 | 210,463 | 0.0104 | 0.0435 | 0.8112 |
| rs75455863 | T | C | 1.0822 | 0.23 | 2.54E-06 |  | 3,283 | 210,463 | 0.0246 | 0.0812 | 0.7615 |
| rs76001965 | C | A | 0.5546 | 0.1254 | 9.74E-06 |  | 3,283 | 210,463 | 0.0558 | 0.0465 | 0.2304 |
| rs76637516 | A | G | 0.7763 | 0.1681 | 3.85E-06 |  | 3,283 | 210,463 | 0.1762 | 0.0616 | 0.004241 |
| rs78686564 | C | T | 0.673 | 0.142 | 2.15E-06 |  | 3,283 | 210,463 | 0.0238 | 0.0521 | 0.6478 |

SNP:single nucleotide polymorphisms; EA: effect allele; OA: other allele; SE, standard error

**Table S3.2** SNPs from GWAS on non-proliferative background diabetic retinopathy and Type 1 diabetes with renal complications

|  | | | **Exposure (non-proliferative DR)** | | |  | **Outcome (Type 1 diabetes with renal complications)** | | | | |
| --- | --- | --- | --- | --- | --- | --- | --- | --- | --- | --- | --- |
| **SNP** | **EA** | **OA** | **β** | **SE** | ***P* value** |  | **Case** | **Control** | **β** | **SE** | ***P* value** |
| rs10875648 | A | G | 0.3278 | 0.0699 | 2.72E-06 |  | 963 | 183,185 | 0.1029 | 0.0484 | 0.03354 |
| rs10948120 | A | G | 0.4408 | 0.0812 | 5.74E-08 |  | 963 | 183,185 | 0.013 | 0.0553 | 0.8146 |
| rs11199244 | A | G | 2.0551 | 0.4567 | 6.80E-06 |  | 963 | 183,185 | 0.3366 | 0.2707 | 0.2137 |
| rs117187956 | A | T | 1.2491 | 0.2605 | 1.63E-06 |  | 963 | 183,185 | 0.2742 | 0.1641 | 0.0948 |
| rs144819595 | T | G | 2.1175 | 0.4703 | 6.72E-06 |  | 963 | 183,185 | 0.2833 | 0.2798 | 0.3114 |
| rs59697139 | G | A | 1.9907 | 0.4487 | 9.14E-06 |  | 963 | 183,185 | 0.5985 | 0.2738 | 0.02885 |
| rs61780990 | T | C | 0.6735 | 0.1522 | 9.60E-06 |  | 963 | 183,185 | 0.1956 | 0.1014 | 0.05368 |
| rs71590933 | A | T | 2.8221 | 0.6321 | 8.01E-06 |  | 963 | 183,185 | 0.3799 | 0.3638 | 0.2964 |
| rs73782012 | C | T | 1.1805 | 0.265 | 8.40E-06 |  | 963 | 183,185 | 0.1912 | 0.1685 | 0.2565 |
| rs75372877 | T | A | 0.5384 | 0.116 | 3.48E-06 |  | 963 | 183,185 | 0.0183 | 0.0796 | 0.818 |
| rs75455863 | T | C | 1.0822 | 0.23 | 2.54E-06 |  | 963 | 183,185 | 0.2248 | 0.1483 | 0.1294 |
| rs76001965 | C | A | 0.5546 | 0.1254 | 9.74E-06 |  | 963 | 183,185 | 4.00E-04 | 0.085 | 0.9959 |
| rs76637516 | A | G | 0.7763 | 0.1681 | 3.85E-06 |  | 963 | 183,185 | 0.2966 | 0.1121 | 0.008159 |
| rs78686564 | C | T | 0.673 | 0.142 | 2.15E-06 |  | 963 | 183,185 | 0.1562 | 0.0956 | 0.1023 |

SNP:single nucleotide polymorphisms; EA: effect allele; OA: other allele; SE, standard error

**Table S3.3** SNPs from GWAS on non-proliferative background diabetic retinopathy and Type 2 diabetes with renal complications

|  | | | **Exposure (non-proliferative DR)** | | |  | **Outcome (Type 2 diabetes with renal complications)** | | | | |
| --- | --- | --- | --- | --- | --- | --- | --- | --- | --- | --- | --- |
| **SNP** | **EA** | **OA** | **β** | **SE** | ***P* value** |  | **Case** | **Control** | **β** | **SE** | ***P* value** |
| rs10875648 | A | G | 0.3278 | 0.0699 | 2.72E-06 |  | 1,296 | 183,185 | 0.067 | 0.0421 | 0.1116 |
| rs10891210 | T | G | -0.3962 | 0.0796 | 6.52E-07 |  | 1,296 | 183,185 | 0.0323 | 0.0483 | 0.5032 |
| rs10948120 | A | G | 0.4408 | 0.0812 | 5.74E-08 |  | 1,296 | 183,185 | -0.0355 | 0.0485 | 0.4642 |
| rs11199244 | A | G | 2.0551 | 0.4567 | 6.80E-06 |  | 1,296 | 183,185 | 0.4934 | 0.239 | 0.03901 |
| rs114496976 | G | T | 1.6411 | 0.3698 | 9.08E-06 |  | 1,296 | 183,185 | -0.0068 | 0.2097 | 0.9743 |
| rs117187956 | A | T | 1.2491 | 0.2605 | 1.63E-06 |  | 1,296 | 183,185 | -0.0966 | 0.1405 | 0.4917 |
| rs117427128 | G | T | 2.4939 | 0.5307 | 2.61E-06 |  | 1,296 | 183,185 | -0.1073 | 0.2532 | 0.6718 |
| rs144819595 | T | G | 2.1175 | 0.4703 | 6.72E-06 |  | 1,296 | 183,185 | -0.0297 | 0.2589 | 0.9088 |
| rs4121484 | T | G | 0.327 | 0.0685 | 1.79E-06 |  | 1,296 | 183,185 | 0.0531 | 0.0413 | 0.1985 |
| rs570170065 | C | T | 1.1285 | 0.2463 | 4.60E-06 |  | 1,296 | 183,185 | 0.2337 | 0.1406 | 0.09639 |
| rs59697139 | G | A | 1.9907 | 0.4487 | 9.14E-06 |  | 1,296 | 183,185 | -0.1622 | 0.2487 | 0.5142 |
| rs61780990 | T | C | 0.6735 | 0.1522 | 9.60E-06 |  | 1,296 | 183,185 | -0.0514 | 0.0885 | 0.5613 |
| rs71590933 | A | T | 2.8221 | 0.6321 | 8.01E-06 |  | 1,296 | 183,185 | -0.191 | 0.3356 | 0.5692 |
| rs73782012 | C | T | 1.1805 | 0.265 | 8.40E-06 |  | 1,296 | 183,185 | 0.1212 | 0.1484 | 0.4139 |
| rs75372877 | T | A | 0.5384 | 0.116 | 3.48E-06 |  | 1,296 | 183,185 | 0.0528 | 0.0696 | 0.4479 |
| rs75455863 | T | C | 1.0822 | 0.23 | 2.54E-06 |  | 1,296 | 183,185 | 0.2784 | 0.1295 | 0.03161 |
| rs76001965 | C | A | 0.5546 | 0.1254 | 9.74E-06 |  | 1,296 | 183,185 | 0.0812 | 0.074 | 0.2726 |
| rs76637516 | A | G | 0.7763 | 0.1681 | 3.85E-06 |  | 1,296 | 183,185 | 0.1658 | 0.0988 | 0.093261 |
| rs78686564 | C | T | 0.673 | 0.142 | 2.15E-06 |  | 1,296 | 183,185 | 0.01 | 0.0831 | 0.9043 |
| rs8192575 | G | C | 1.2349 | 0.2024 | 1.04E-09 |  | 1,296 | 183,185 | 0.3014 | 0.1172 | 0.01011 |

SNP:single nucleotide polymorphisms; EA: effect allele; OA: other allele; SE, standard error

**Table S3.4** SNPs from GWAS on non-proliferative background diabetic retinopathy and Glomerular filtration rate in diabetics

|  | | | **Exposure (non-proliferative DR)** | | |  | **Outcome (Glomerular filtration rate in diabetics)** | | | | |
| --- | --- | --- | --- | --- | --- | --- | --- | --- | --- | --- | --- |
| **SNP** | **EA** | **OA** | **β** | **SE** | ***P* value** |  | **Case** | **Control** | **β** | **SE** | ***P* value** |
| rs10875648 | A | G | 0.3278 | 0.0699 | 2.72E-06 |  | 11,522 | 133,413 | -0.0048 | 0.0038 | 0.21 |
| rs10891210 | T | G | -0.3962 | 0.0796 | 6.52E-07 |  | 11,522 | 133,413 | -0.0016 | 0.0061 | 0.79 |
| rs8192575 | G | C | 1.2349 | 0.2024 | 1.04E-09 |  | 11,522 | 133,413 | -0.005 | 0.0083 | 0.55 |

SNP:single nucleotide polymorphisms; EA: effect allele; OA: other allele; SE, standard error

**Table S3.5** SNPs from GWAS on non-proliferative background diabetic retinopathy and albumin-to-creatinine ratio in diabetics

|  | | | **Exposure (non-proliferative DR)** | | |  | **Outcome (** **albumin-to-creatinine ratio in diabetics )** | | | | |
| --- | --- | --- | --- | --- | --- | --- | --- | --- | --- | --- | --- |
| **SNP** | **EA** | **OA** | **β** | **SE** | ***P* value** |  | **Case** | **Control** | **β** | **SE** | ***P* value** |
| rs10875648 | A | G | 0.3278 | 0.0699 | 2.72E-06 |  | 5,825 | 46061 | 0.065 | 0.03 | 0.032 |
| rs10891210 | T | G | -0.3962 | 0.0796 | 6.52E-07 |  | 5,825 | 46061 | -0.03 | 0.046 | 0.52 |
| rs8192575 | G | C | 1.2349 | 0.2024 | 1.04E-09 |  | 5,825 | 46061 | -0.04 | 0.069 | 0.56 |

SNP:single nucleotide polymorphisms; EA: effect allele; OA: other allele; SE, standard error

**Table S4.1 SNPs from GWAS on proliferative background diabetic retinopathy and Diabetic nephropathy**

|  | | | **Exposure (proliferative DR)** | | |  | **Outcome (Diabetic nephropathy)** | | | | |
| --- | --- | --- | --- | --- | --- | --- | --- | --- | --- | --- | --- |
| **SNP** | **EA** | **OA** | **β** | **SE** | ***P* value** |  | **Case** | **Control** | **β** | **SE** | ***P* value** |
| rs11243147 | T | C | 0.0921 | 0.0166 | 3.02E-08 |  | 3,283 | 210,463 | 0.0369 | 0.0259 | 0.155 |
| rs114379727 | A | G | 0.3426 | 0.0515 | 2.86E-11 |  | 3,283 | 210,463 | 0.2742 | 0.0802 | 0.000628 |
| rs114785141 | T | C | 0.4671 | 0.1043 | 7.59E-06 |  | 3,283 | 210,463 | 0.4793 | 0.163 | 0.003273 |
| rs116919772 | T | G | 0.1459 | 0.0327 | 8.20E-06 |  | 3,283 | 210,463 | 0.044 | 0.0508 | 0.3872 |
| rs11774990 | T | C | 0.0919 | 0.0204 | 7.03E-06 |  | 3,283 | 210,463 | 0.0101 | 0.0319 | 0.7527 |
| rs12507762 | C | T | -0.0749 | 0.0169 | 9.65E-06 |  | 3,283 | 210,463 | -0.0126 | 0.0264 | 0.6326 |
| rs144260139 | A | G | 0.332 | 0.072 | 4.07E-06 |  | 3,283 | 210,463 | 0.1358 | 0.1124 | 0.227 |
| rs145526532 | G | A | 0.2199 | 0.0465 | 2.27E-06 |  | 3,283 | 210,463 | 0.0496 | 0.0723 | 0.4924 |
| rs148086391 | T | C | 0.2149 | 0.0485 | 9.44E-06 |  | 3,283 | 210,463 | 0.1834 | 0.0759 | 0.01565 |
| rs16968859 | G | A | -0.1097 | 0.0234 | 2.70E-06 |  | 3,283 | 210,463 | 0.0258 | 0.0364 | 0.4783 |
| rs2523953 | A | G | -0.1907 | 0.0262 | 3.61E-13 |  | 3,283 | 210,463 | -0.1033 | 0.0403 | 0.01039 |
| rs2597318 | G | A | -0.0736 | 0.0166 | 9.80E-06 |  | 3,283 | 210,463 | -0.0457 | 0.0259 | 0.07792 |
| rs34106814 | T | C | -0.1402 | 0.0312 | 6.81E-06 |  | 3,283 | 210,463 | -0.0285 | 0.0483 | 0.554501 |
| rs35695736 | A | G | -0.094 | 0.0208 | 6.52E-06 |  | 3,283 | 210,463 | -0.0712 | 0.0325 | 0.02837 |
| rs4502225 | C | T | -0.1201 | 0.0247 | 1.19E-06 |  | 3,283 | 210,463 | -0.0669 | 0.0386 | 0.08358 |
| rs6509507 | A | G | 0.1022 | 0.0215 | 2.06E-06 |  | 3,283 | 210,463 | 0.0039 | 0.0337 | 0.9072 |
| rs710583 | C | A | 0.089 | 0.0172 | 2.16E-07 |  | 3,283 | 210,463 | 0.0444 | 0.0268 | 0.097011 |
| rs7260507 | C | A | -0.0785 | 0.017 | 3.88E-06 |  | 3,283 | 210,463 | -0.0483 | 0.0265 | 0.06839 |
| rs77873003 | T | C | 0.1895 | 0.0425 | 8.19E-06 |  | 3,283 | 210,463 | 0.1543 | 0.0663 | 0.02 |
| rs7805376 | G | T | -0.075 | 0.0167 | 7.35E-06 |  | 3,283 | 210,463 | -0.0535 | 0.0261 | 0.04047 |
| rs8059028 | A | C | 0.0777 | 0.0169 | 4.30E-06 |  | 3,283 | 210,463 | 0.0767 | 0.0264 | 0.003616 |
| rs9366187 | C | G | 0.0908 | 0.0192 | 2.37E-06 |  | 3,283 | 210,463 | 0.0584 | 0.03 | 0.05173 |
| rs9394159 | T | A | 0.0854 | 0.0164 | 1.91E-07 |  | 3,283 | 210,463 | 0.061 | 0.0257 | 0.0176 |

SNP:single nucleotide polymorphisms; EA: effect allele; OA: other allele; SE, standard error

**Table S4.2 SNPs from GWAS on proliferative background diabetic retinopathy and Type 1 diabetes with renal complications**

|  | | | **Exposure (proliferative DR)** | | |  | **Outcome (Type 1 diabetes with renal complications)** | | | | |
| --- | --- | --- | --- | --- | --- | --- | --- | --- | --- | --- | --- |
| **SNP** | **EA** | **OA** | **β** | **SE** | ***P* value** |  | **Case** | **Control** | **β** | **SE** | ***P* value** |
| rs11243147 | T | C | 0.0921 | 0.0166 | 3.02E-08 |  | 963 | 183,185 | 0.0391 | 0.0475 | 0.4108 |
| rs114785141 | T | C | 0.4671 | 0.1043 | 7.59E-06 |  | 963 | 183,185 | 0.6809 | 0.2954 | 0.02116 |
| rs11774990 | T | C | 0.0919 | 0.0204 | 7.03E-06 |  | 963 | 183,185 | -0.0124 | 0.0588 | 0.8326 |
| rs12507762 | C | T | -0.0749 | 0.0169 | 9.65E-06 |  | 963 | 183,185 | -0.0057 | 0.0481 | 0.9065 |
| rs144260139 | A | G | 0.332 | 0.072 | 4.07E-06 |  | 963 | 183,185 | 0.3491 | 0.2032 | 0.08588 |
| rs145526532 | G | A | 0.2199 | 0.0465 | 2.27E-06 |  | 963 | 183,185 | 0.2339 | 0.1315 | 0.075211 |
| rs148086391 | T | C | 0.2149 | 0.0485 | 9.44E-06 |  | 963 | 183,185 | 0.3094 | 0.1409 | 0.02805 |
| rs16968859 | G | A | -0.1097 | 0.0234 | 2.70E-06 |  | 963 | 183,185 | -0.0087 | 0.0667 | 0.8965 |
| rs2523953 | A | G | -0.1907 | 0.0262 | 3.61E-13 |  | 963 | 183,185 | -0.4299 | 0.076 | 1.54E-08 |
| rs2597318 | G | A | -0.0736 | 0.0166 | 9.80E-06 |  | 963 | 183,185 | -0.1469 | 0.0474 | 0.00196 |
| rs34106814 | T | C | -0.1402 | 0.0312 | 6.81E-06 |  | 963 | 183,185 | -0.0461 | 0.0879 | 0.600101 |
| rs35695736 | A | G | -0.094 | 0.0208 | 6.52E-06 |  | 963 | 183,185 | -0.1408 | 0.0593 | 0.01765 |
| rs4502225 | C | T | -0.1201 | 0.0247 | 1.19E-06 |  | 963 | 183,185 | -0.1474 | 0.0708 | 0.03743 |
| rs6509507 | A | G | 0.1022 | 0.0215 | 2.06E-06 |  | 963 | 183,185 | 0.0675 | 0.0615 | 0.2725 |
| rs710583 | C | A | 0.089 | 0.0172 | 2.16E-07 |  | 963 | 183,185 | 0.1485 | 0.049 | 0.002446 |
| rs7260507 | C | A | -0.0785 | 0.017 | 3.88E-06 |  | 963 | 183,185 | -0.0914 | 0.0486 | 0.05975 |
| rs77873003 | T | C | 0.1895 | 0.0425 | 8.19E-06 |  | 963 | 183,185 | 0.2843 | 0.121 | 0.01883 |
| rs7805376 | G | T | -0.075 | 0.0167 | 7.35E-06 |  | 963 | 183,185 | -0.0829 | 0.0479 | 0.08348 |
| rs7903146 | T | C | 0.1137 | 0.0205 | 2.82E-08 |  | 963 | 183,185 | 0.1305 | 0.0587 | 0.02617 |
| rs8059028 | A | C | 0.0777 | 0.0169 | 4.30E-06 |  | 963 | 183,185 | 0.0854 | 0.0482 | 0.076551 |
| rs9366187 | C | G | 0.0908 | 0.0192 | 2.37E-06 |  | 963 | 183,185 | 0.0379 | 0.0549 | 0.4903 |
| rs9394159 | T | A | 0.0854 | 0.0164 | 1.91E-07 |  | 963 | 183,185 | 0.2275 | 0.047 | 1.32E-06 |

SNP:single nucleotide polymorphisms; EA: effect allele; OA: other allele; SE, standard error

**Table S4.3 SNPs from GWAS on proliferative background diabetic retinopathy and Type 2 diabetes with renal complications**

|  | | | **Exposure (proliferative DR)** | | |  | **Outcome (Type 2 diabetes with renal complications)** | | | | |
| --- | --- | --- | --- | --- | --- | --- | --- | --- | --- | --- | --- |
| **SNP** | **EA** | **OA** | **β** | **SE** | ***P* value** |  | **Case** | **Control** | **β** | **SE** | ***P* value** |
| rs11243147 | T | C | 0.0921 | 0.0166 | 3.02E-08 |  | 1,296 | 183,185 | 0.0462 | 0.0415 | 0.2656 |
| rs114379727 | A | G | 0.3426 | 0.0515 | 2.86E-11 |  | 1,296 | 183,185 | 0.0702 | 0.1293 | 0.5874 |
| rs114785141 | T | C | 0.4671 | 0.1043 | 7.59E-06 |  | 1,296 | 183,185 | 0.588 | 0.265 | 0.02649 |
| rs116919772 | T | G | 0.1459 | 0.0327 | 8.20E-06 |  | 1,296 | 183,185 | 0.1063 | 0.0803 | 0.1854 |
| rs11774990 | T | C | 0.0919 | 0.0204 | 7.03E-06 |  | 1,296 | 183,185 | 0.0247 | 0.051 | 0.6275 |
| rs12507762 | C | T | -0.0749 | 0.0169 | 9.65E-06 |  | 1,296 | 183,185 | 0.035 | 0.0424 | 0.4088 |
| rs144260139 | A | G | 0.332 | 0.072 | 4.07E-06 |  | 1,296 | 183,185 | 0.0202 | 0.1799 | 0.9108 |
| rs145526532 | G | A | 0.2199 | 0.0465 | 2.27E-06 |  | 1,296 | 183,185 | -0.0583 | 0.1163 | 0.6163 |
| rs148086391 | T | C | 0.2149 | 0.0485 | 9.44E-06 |  | 1,296 | 183,185 | 0.447 | 0.1231 | 0.000283 |
| rs16968859 | G | A | -0.1097 | 0.0234 | 2.70E-06 |  | 1,296 | 183,185 | -0.0077 | 0.0581 | 0.8953 |
| rs2523953 | A | G | -0.1907 | 0.0262 | 3.61E-13 |  | 1,296 | 183,185 | -0.0853 | 0.0636 | 0.1803 |
| rs2597318 | G | A | -0.0736 | 0.0166 | 9.80E-06 |  | 1,296 | 183,185 | -0.071 | 0.0413 | 0.08599 |
| rs34106814 | T | C | -0.1402 | 0.0312 | 6.81E-06 |  | 1,296 | 183,185 | -0.04 | 0.0764 | 0.6002 |
| rs34337125 | A | G | -0.0751 | 0.0167 | 6.86E-06 |  | 1,296 | 183,185 | -0.029 | 0.0414 | 0.4831 |
| rs35695736 | A | G | -0.094 | 0.0208 | 6.52E-06 |  | 1,296 | 183,185 | -0.0697 | 0.0518 | 0.1784 |
| rs4502225 | C | T | -0.1201 | 0.0247 | 1.19E-06 |  | 1,296 | 183,185 | -0.1285 | 0.0619 | 0.03795 |
| rs6509507 | A | G | 0.1022 | 0.0215 | 2.06E-06 |  | 1,296 | 183,185 | 0.0865 | 0.0535 | 0.1059 |
| rs710583 | C | A | 0.089 | 0.0172 | 2.16E-07 |  | 1,296 | 183,185 | 0.0775 | 0.0428 | 0.07012 |
| rs7260507 | C | A | -0.0785 | 0.017 | 3.88E-06 |  | 1,296 | 183,185 | -0.0194 | 0.0423 | 0.646399 |
| rs77873003 | T | C | 0.1895 | 0.0425 | 8.19E-06 |  | 1,296 | 183,185 | 0.3277 | 0.1075 | 0.002294 |
| rs7805376 | G | T | -0.075 | 0.0167 | 7.35E-06 |  | 1,296 | 183,185 | -0.0452 | 0.0416 | 0.2775 |
| rs8059028 | A | C | 0.0777 | 0.0169 | 4.30E-06 |  | 1,296 | 183,185 | 0.0783 | 0.0419 | 0.061669 |
| rs9366187 | C | G | 0.0908 | 0.0192 | 2.37E-06 |  | 1,296 | 183,185 | 0.0963 | 0.0478 | 0.04394 |
| rs9394159 | T | A | 0.0854 | 0.0164 | 1.91E-07 |  | 1,296 | 183,185 | -0.0189 | 0.041 | 0.645199 |

SNP:single nucleotide polymorphisms; EA: effect allele; OA: other allele; SE, standard error

**Table S4.4** SNPs from GWAS on proliferative background diabetic retinopathy and Glomerular filtration rate in diabetics

|  | | | **Exposure (proliferative DR)** | | |  | **Outcome (Glomerular filtration rate in diabetics)** | | | | |
| --- | --- | --- | --- | --- | --- | --- | --- | --- | --- | --- | --- |
| **SNP** | **EA** | **OA** | **β** | **SE** | ***P* value** |  | **Case** | **Control** | **β** | **SE** | ***P* value** |
| rs11243147 | T | C | 0.0921 | 0.0166 | 3.02E-08 |  | 11,522 | 133,413 | 0.0016 | 0.0037 | 0.67 |
| rs11774990 | T | C | 0.0919 | 0.0204 | 7.03E-06 |  | 11,522 | 133,413 | -0.0053 | 0.0048 | 0.27 |
| rs2597318 | G | A | -0.0736 | 0.0166 | 9.80E-06 |  | 11,522 | 133,413 | 9.00E-04 | 0.0036 | 0.8 |
| rs4502225 | C | T | -0.1201 | 0.0247 | 1.19E-06 |  | 11,522 | 133,413 | 0.0025 | 0.0067 | 0.709999 |
| rs6509507 | A | G | 0.1022 | 0.0215 | 2.06E-06 |  | 11,522 | 133,413 | 0.012 | 0.0054 | 0.028 |
| rs7260507 | C | A | -0.0785 | 0.017 | 3.88E-06 |  | 11,522 | 133,413 | 0.0061 | 0.0045 | 0.17 |
| rs7805376 | G | T | -0.075 | 0.0167 | 7.35E-06 |  | 11,522 | 133,413 | -0.0028 | 0.0036 | 0.44 |
| rs8059028 | A | C | 0.0777 | 0.0169 | 4.30E-06 |  | 11,522 | 133,413 | -0.0017 | 0.0038 | 0.66 |
| rs9394159 | T | A | 0.0854 | 0.0164 | 1.91E-07 |  | 11,522 | 133,413 | 0.0053 | 0.0036 | 0.14 |

SNP:single nucleotide polymorphisms; EA: effect allele; OA: other allele; SE, standard error

**Table S4.5** SNPs from GWAS on proliferative background diabetic retinopathy and albumin-to-creatinine ratio in diabetics

|  | | | **Exposure (proliferative DR)** | | |  | **Outcome (** **albumin-to-creatinine ratio in diabetics )** | | | | |
| --- | --- | --- | --- | --- | --- | --- | --- | --- | --- | --- | --- |
| **SNP** | **EA** | **OA** | **β** | **SE** | ***P* value** |  | **Case** | **Control** | **β** | **SE** | ***P* value** |
| rs11774990 | T | C | 0.0919 | 0.0204 | 7.03E-06 |  | 5,825 | 46061 | 0.0025 | 0.037 | 0.95 |
| rs4502225 | C | T | -0.1201 | 0.0247 | 1.19E-06 |  | 5,825 | 46061 | -0.0077 | 0.051 | 0.88 |
| rs6509507 | A | G | 0.1022 | 0.0215 | 2.06E-06 |  | 5,825 | 46061 | 0.006 | 0.045 | 0.89 |
| rs7260507 | C | A | -0.0785 | 0.017 | 3.88E-06 |  | 5,825 | 46061 | 0.016 | 0.039 | 0.69 |
| rs8059028 | A | C | 0.0777 | 0.0169 | 4.30E-06 |  | 5,825 | 46061 | 0.034 | 0.029 | 0.25 |
| rs9394159 | T | A | 0.0854 | 0.0164 | 1.91E-07 |  | 5,825 | 46061 | -0.045 | 0.029 | 0.12 |

SNP:single nucleotide polymorphisms; EA: effect allele; OA: other allele; SE, standard error

| **Exposure** | **Outcome** | **MR Methods** | **SNPs** | **beta** | **P value** |
| --- | --- | --- | --- | --- | --- |
|  | Diabetic Nephropathy | MR Egger | 19 | 0.057778 | 1.02E-01 |
|  |  | Weighted median | 19 | 0.023967 | 3.35E-01 |
|  |  | Inverse variance weighted | 19 | 0.064439 | 3.96E-04 |
|  |  | Simple mode | 19 | 0.014028 | 7.46E-01 |
|  |  | Weighted mode | 19 | 0.01324 | 7.31E-01 |
|  | Type 1 diabetes with renal complications | MR Egger | 14 | 0.19909 | 1.48E-02 |
|  |  | Weighted median | 14 | 0.163581 | 2.18E-03 |
|  |  | Inverse variance weighted | 14 | 0.183241 | 7.71E-07 |
|  |  | Simple mode | 14 | 0.193587 | 5.70E-02 |
|  |  | Weighted mode | 14 | 0.180437 | 6.00E-02 |
|  | Type 2 diabetes with renal complications | MR Egger | 20 | 0.053392 | 3.46E-01 |
| Non-proliferative DR |  | Weighted median | 20 | 0.010603 | 7.98E-01 |
|  |  | Inverse variance weighted | 20 | 0.068365 | 2.29E-02 |
|  |  | Simple mode | 20 | -0.05244 | 5.75E-01 |
|  |  | Weighted mode | 20 | -0.05244 | 5.64E-01 |
|  | Glomerular filtration rate | MR Egger | 3 | -0.00138 | 9.13E-01 |
|  |  | Weighted median | 3 | -0.00505 | 4.12E-01 |
|  |  | Inverse variance weighted | 3 | -0.00537 | 3.23E-01 |
|  |  | Simple mode | 3 | -0.00223 | 8.10E-01 |
|  |  | Weighted mode | 3 | -0.00432 | 5.93E-01 |
|  | Urinary albumin-to-creatinine ratio | MR Egger | 3 | -0.1109 | 4.07E-01 |
|  |  | Weighted median | 3 | 0.015055 | 7.71E-01 |
|  |  | Inverse variance weighted | 3 | 0.036811 | 5.89E-01 |
|  |  | Simple mode | 3 | 0.062208 | 6.30E-01 |
|  |  | Weighted mode | 3 | -0.01933 | 7.53E-01 |
|  | Diabetic Nephropathy | MR Egger | 22 | 0.643896 | 8.62E-04 |
|  |  | Weighted median | 22 | 0.533031 | 2.97E-07 |
|  |  | Inverse variance weighted | 22 | 0.511651 | 3.70E-14 |
|  |  | Simple mode | 22 | 0.631129 | 2.21E-03 |
|  |  | Weighted mode | 22 | 0.574916 | 3.07E-03 |
|  | Type 1 diabetes with renal complications | MR Egger | 21 | 1.653796 | 5.12E-04 |
|  |  | Weighted median | 21 | 1.101829 | 2.15E-08 |
|  |  | Inverse variance weighted | 21 | 1.093766 | 1.95E-13 |
|  |  | Simple mode | 21 | 1.240628 | 4.69E-03 |
|  |  | Weighted mode | 21 | 1.208463 | 9.21E-03 |
|  | Type 2 diabetes with renal complications | MR Egger | 23 | 0.648952 | 3.65E-02 |
|  |  | Weighted median | 23 | 0.470464 | 4.88E-03 |
| Proliferative DR |  | Inverse variance weighted | 23 | 0.602169 | 1.81E-07 |
|  |  | Simple mode | 23 | 0.457744 | 8.78E-02 |
|  |  | Weighted mode | 23 | 0.393629 | 1.03E-01 |
|  | Glomerular filtration rate | MR Egger | 8 | 0.086922 | 5.77E-01 |
|  |  | Weighted median | 8 | -0.01256 | 5.89E-01 |
|  |  | Inverse variance weighted | 8 | 0.001016 | 9.60E-01 |
|  |  | Simple mode | 8 | -0.01671 | 6.65E-01 |
|  |  | Weighted mode | 8 | 0.0067 | 8.42E-01 |
|  | Urinary albumin-to-creatinine ratio | MR Egger | 5 | -0.30897 | 8.14E-01 |
|  |  | Weighted median | 5 | 0.059766 | 7.97E-01 |
|  |  | Inverse variance weighted | 5 | 0.111792 | 5.53E-01 |
|  |  | Simple mode | 5 | 0.051555 | 8.81E-01 |
|  |  | Weighted mode | 5 | 0.051555 | 8.76E-01 |

**Table S5** MR results for association of non-proliferative DR and proliferative DR with DN.

SNP:single nucleotide polymorphisms**.**

**Table S6** Heterogeneity of MR analysis for DR and DN.

| **Exposure** | **Outcome** | **MR Methods** | **Cochran Q statistic** | **Q_df** | | | **Q_pval** |
| --- | --- | --- | --- | --- | --- | --- | --- |
| Background diabetic retinopathy | Diabetic Nephropathy | MR Egger | 5.748634 | 12 | | | 0.9282192 |
|  |  | Inverse variance weighted | 6.032175 | 13 | | | 0.9449741 |
|  | Type 1 diabetes with renal complications | MR Egger | 4.445928 | 11 | | | 0.9550145 |
|  |  | Inverse variance weighted | 4.445946 | 12 | | | 0.9739810 |
|  | Type 2 diabetes with renal complications | MR Egger | 12.75753 | 13 | | | 0.4667104 |
|  |  | Inverse variance weighted | 12.7601 | 14 | | | 0.5454946 |
|  | Glomerular filtration rate | Inverse variance weighted | 0.01094892 | 1 | | | 0.9166638 |
|  | Urinary albumin-to-creatinine ratio | Inverse variance weighted | 0.4763597 | 1 | | | 0.4900758 |
| Non-proliferative diabetic retinopathy | Diabetic Nephropathy | MR Egger | 19.58769 | 17 | | | 0.2958595 |
|  |  | Inverse variance weighted | 19.65415 | 18 | | | 0.3526234 |
|  | Type 1 diabetes with renal complications | MR Egger | 8.335546 | 12 | | | 0.7583897 |
|  |  | Inverse variance weighted | 8.406636 | 13 | | | 0.8161373 |
|  | Type 2 diabetes with renal complications | MR Egger | 23.7934 | 18 | | | 0.1619135 |
|  |  | Inverse variance weighted | 23.93466 | 19 | | | 0.1986708 |
|  | Glomerular filtration rate | MR Egger | 0.8239534 | 1 | | | 0.3640268 |
|  |  | Inverse variance weighted | 1.0519336 | 2 | | | 0.5909837 |
|  | Urinary albumin-to-creatinine ratio | MR Egger | 0.2518352 | 1 | | | 0.61578581 |
|  |  | Inverse variance weighted | 4.7595042 | 2 | | | 0.09257353 |
| Proliferative diabetic retinopathy | Diabetic Nephropathy | MR Egger | 19.37957 | 20 | | | 0.4972907 |
|  |  | Inverse variance weighted | 20.15643 | 21 | | | 0.5113727 |
|  | Type 1 diabetes with renal complications | MR Egger | 24.04687 | | 19 | 0.1943605 | |
|  |  | Inverse variance weighted | 26.96562 | | 20 | 0.1362339 | |
|  | Type 2 diabetes with renal complications | MR Egger | 26.04158 | | 21 | 0.2048679 | |
|  |  | Inverse variance weighted | 26.08011 | | 22 | 0.2482583 | |

**Table S7** horizontal pleiotropy test of MR analysis for DR and DN.

| **Exposure** | **Outcome** | **MR Methods** | **Egger intercept** | **SE** | **Pleiotropy p-value** |
| --- | --- | --- | --- | --- | --- |
| Background diabetic retinopathy | Diabetic Nephropathy | MR Egger | -0.0119576 | 0.02245618 | 0.6041098 |
|  |  | MR Presso |  |  | 0.9614 |
|  | Type 1 diabetes with renal complications | MR Egger | 0.0001045037 | 0.02481867 | 0.9967158 |
|  |  | MR Presso |  |  | 0.9876667 |
|  | Type 2 diabetes with renal complications | MR Egger | -0.001693133 | 0.03340995 | 0.9603529 |
|  |  | MR Presso |  |  | 0.5636667 |
| Non-proliferative diabetic retinopathy | Diabetic Nephropathy | MR Egger | 0.0052106 | 0.02169509 | 0.8130674 |
|  |  | MR Presso |  |  | 0.3956667 |
|  | Type 1 diabetes with renal complications | MR Egger | -0.01300252 | 0.04876669 | 0.7942827 |
|  |  | MR Presso |  |  | 0.8676667 |
|  | Type 2 diabetes with renal complications | MR Egger | 0.01202139 | 0.03677427 | 0.7475167 |
|  |  | MR Presso |  |  | 0.2046667 |
|  | Glomerular filtration rate | MR Egger | -0.002634323 | 0.005517223 | 0.7164089 |
|  | Urinary albumin-to-creatinine ratio | MR Egger | 0.09361623 | 0.04409356 | 0.2802288 |
| Proliferative diabetic retinopathy | Diabetic Nephropathy | MR Egger | -0.01609629 | 0.01826222 | 0.3885723 |
|  |  | MR Presso |  |  | 0.6417 |
|  | Type 1 diabetes with renal complications | MR Egger | -0.06371645 | 0.04195711 | 0.1453258 |
|  |  | MR Presso |  |  | 0.23 |
|  | Type 2 diabetes with renal complications | MR Egger | -0.005553915 | 0.03150865 | 0.8617735 |
|  |  | MR Presso |  |  | 0.3243333 |
|  | Glomerular filtration rate | MR Egger | -0.007398813 | 0.01255757 | 0.5772276 |
|  |  | MR Presso |  |  | 0.269 |
|  | Urinary albumin-to-creatinine ratio | MR Egger | 0.03841961 | 0.1087002 | 0.747133 |
|  |  | MR Presso |  |  | 0.8736667 |

Table S8.1 Multivariable MR estimates for the causal effect of genetical proxied DR, non-proliferative DR, proliferative DR (adjusted for HbA1C and fasting glucose).

| **Exposure** | **Outcome** | **MR Methods** | **SNPs** | **OR (95%CI)** | **P value** | **Pleiotropy p-value** | | |
| --- | --- | --- | --- | --- | --- | --- | --- | --- |
|  |  |  |  |  |  | **MR Presso** | **MR Egger** | |
| Background diabetic retinopathy, adjust for  HbA1C | Diabetic Nephropathy | IVW | 3 | 1.57 (1.47-1.68) | 2.98E-43 | 0.055 | | 0.061 |
|  | Type 1 diabetes with renal complications | IVW | 3 | 3.66(3.13-4.29) | 1.70E-58 | 0.042 | | 0.018 |
|  | Type 2 diabetes with renal complications | IVW | 3 | 1.22(1.11-1.34) | 3.87E-05 | 0.087 | | 0.779 |
|  | Urinary albumin-to-creatinine ratio | IVW | 3 | 0.95(0.90-1.00) | 5.70E-02 | 0.552 | | 0.259 |
| Background diabetic retinopathy, adjust for  fasting glucose | Diabetic Nephropathy | IVW | 1 | 1.54(1.34-1.76) | 4.94E-10 | 0.505 | | 0.273 |
|  | Type 1 diabetes with renal complications | IVW | 1 | 2.89(2.29-3.64) | 3.12E-19 | 0.721 | | 0.002 |
|  | Type 2 diabetes with renal complications | IVW | 1 | 1.49(1.18-1.88) | 7.89E-04 | 0.101 | | 0.162 |
|  | Urinary albumin-to-creatinine ratio | IVW | 1 | 0.95(0.80-1.11) | 4.97E-01 | 0.111 | | 0.838 |
| non-proliferative DR, adjust for HbA1C | Diabetic Nephropathy | IVW | 1 | 1.50(1.35-1.65) | 2.72E-15 | 0.129 | | 0.003 |
|  | Type 1 diabetes with renal complications | IVW | 1 | 2.11(1.65-2.69) | 2.22E-09 | 0.441 | | 0.062 |
|  | Type 2 diabetes with renal complications | IVW | 1 | 1.11(0.98-1.25) | 6.36E-02 | 1.439 | | 0.289 |
| non-proliferative DR, adjust for fasting glucose | Diabetic Nephropathy | IVW | NA | NA | NA | NA | | NA |
|  | Type 1 diabetes with renal complications | IVW | NA | NA | NA | NA | | NA |
|  | Type 2 diabetes with renal complications | IVW | NA | NA | NA | NA | | NA |
| Proliferative DR, adjust for HbA1C | Diabetic Nephropathy | IVW | 6 | 2.13(1.96-2.31) | 4.27E-21 | 0.432 | | 0.402 |
|  | Type 1 diabetes with renal complications | IVW | 6 | 8.17(6.55-10.20) | 1.19E-26 | 0.543 | | 0.024 |
|  | Type 2 diabetes with renal complications | IVW | 6 | 1.50(1.31-1.72) | 4.65E-09 | 0.191 | | 0.758 |
| Proliferative DR, adjust for fasting glucose | Diabetic Nephropathy | IVW | 2 | 2.16(1.70-2.73) | 2.45E-10 | 0.328 | | 0.259 |
|  | Type 1 diabetes with renal complications | IVW | 2 | 5.96(3.95-9.00) | 2.07E-17 | 0.469 | | 0.167 |
|  | Type 2 diabetes with renal complications | IVW | 2 | 2.44(1.68-3.54) | 2.79E-06 | 0.189 | | 0.376 |

Table S8.2 Variants Used as Instruments for Multivariable MR estimates for the causal effect of DR (adjusted for HbA1C

| **SNP** | **Effect Allele** | **Other Allele** | **Association with HbA1C** | | | **Association with Diabetic Retinopathy** | | |
| --- | --- | --- | --- | --- | --- | --- | --- | --- |
|  |  |  | **Beta** | **SE** | **P** | **Beta** | **SE** | **P** |
| rs10100688 | A | G | 0.0285 | 0.0057 | 4.826E-07 | -0.0388 | 0.0454 | 0.3933 |
| rs1046896 | T | C | 0.0346 | 0.0032 | 1.5762E-26 | 0.0114 | 0.0373 | 0.760001 |
| rs10806742 | C | T | -0.0215 | 0.0047 | 4.754E-06 | 0.0401 | 0.0342 | 0.2403 |
| rs10934830 | T | G | 0.0696 | 0.0154 | 6.003E-06 | -0.1192 | 0.0748 | 0.1112 |
| rs11964178 | G | A | -0.0168 | 0.0034 | 8.786E-07 | 0.0611 | 0.0328 | 0.0626801 |
| rs12255372 | T | G | 0.0173 | 0.0038 | 5.3599E-06 | 0.0493 | 0.0425 | 0.2453 |
| rs12580246 | A | G | 0.0284 | 0.0061 | 3.755E-06 | 0.0778 | 0.0529 | 0.1409 |
| rs12819124 | A | C | -0.0161 | 0.0034 | 1.831E-06 | -0.0039 | 0.0332 | 0.9062 |
| rs1387153 | T | C | 0.0258 | 0.0039 | 3.961E-11 | 0.0271 | 0.0348 | 0.437 |
| rs16926246 | T | C | -0.089 | 0.0057 | 3.1103E-54 | -0.1215 | 0.0642 | 0.0583996 |
| rs17533945 | C | T | 0.0179 | 0.0038 | 1.933E-06 | -0.0157 | 0.0329 | 0.632501 |
| rs17789266 | T | C | 0.023 | 0.0047 | 8.136E-07 | -0.0463 | 0.0544 | 0.3949 |
| rs1789891 | A | C | -0.0207 | 0.0045 | 4.675E-06 | 0.0228 | 0.0394 | 0.5636 |
| rs1799884 | T | C | 0.038 | 0.0041 | 1.4511E-20 | -0.0572 | 0.0531 | 0.2811 |
| rs1800562 | A | G | -0.0636 | 0.0069 | 2.593E-20 | -0.0214 | 0.0858 | 0.8033 |
| rs2476601 | G | A | -0.0038 | 0.0057 | 0.5053 | -0.4128 | 0.047 | 1.6661E-18 |
| rs26710 | T | C | 0.0031 | 0.0039 | 0.4235 | -0.1797 | 0.0377 | 1.92699E-06 |
| rs2723517 | C | A | -0.0159 | 0.0035 | 4.017E-06 | 0.0403 | 0.0327 | 0.2183 |
| rs2779116 | T | C | 0.0237 | 0.004 | 2.751E-09 | 0.0417 | 0.0348 | 0.2309 |
| rs3006789 | T | C | 0.0168 | 0.0037 | 7.002E-06 | -0.0382 | 0.0349 | 0.2739 |
| rs3782123 | A | C | -0.0201 | 0.0042 | 1.672E-06 | -0.0373 | 0.0383 | 0.3304 |
| rs3957146 | C | T | 0.0041 | 0.0061 | 0.509 | 1.3226 | 0.0538 | 2.6607E-133 |
| rs4737009 | A | G | 0.0269 | 0.0039 | 6.1151E-12 | 0.0179 | 0.0382 | 0.6387 |
| rs4844390 | G | A | -0.0204 | 0.0041 | 6.8991E-07 | -0.0731 | 0.0396 | 0.0650999 |
| rs552976 | G | A | 0.029 | 0.0034 | 8.1564E-18 | 0.0071 | 0.0346 | 0.8373 |
| rs6453220 | T | C | 0.0456 | 0.0099 | 4.186E-06 | 0.0238 | 0.1292 | 0.8539 |
| rs6474359 | C | T | -0.0601 | 0.0105 | 1.178E-08 | -0.0687 | 0.1243 | 0.5802 |
| rs6844670 | G | A | 0.0203 | 0.0039 | 2.123E-07 | 0.0205 | 0.0334 | 0.5396 |
| rs7355559 | T | G | -0.0241 | 0.0052 | 3.862E-06 | -0.1149 | 0.0576 | 0.04617 |
| rs7805661 | C | A | 0.0366 | 0.0079 | 4.09E-06 | 0.0853 | 0.0682 | 0.2112 |
| rs7908691 | G | T | -0.0025 | 0.0035 | 0.4812 | 0.1541 | 0.0348 | 9.49598E-06 |
| rs7998202 | G | A | 0.0307 | 0.0053 | 5.235E-09 | -0.0068 | 0.05 | 0.8914 |
| rs837763 | T | C | 0.0245 | 0.0048 | 2.696E-07 | -0.014 | 0.033 | 0.6705 |
| rs855791 | G | A | -0.0271 | 0.0036 | 2.7422E-14 | -0.0021 | 0.0344 | 0.9506 |
| rs915894 | G | T | 0.0031 | 0.0062 | 0.6158 | 0.5227 | 0.0369 | 1.46994E-45 |
| rs9318651 | T | C | 0.0367 | 0.0079 | 0.00000315 | -0.0644 | 0.0335 | 0.0544703 |
| rs9354939 | C | T | -0.0163 | 0.0035 | 3.1E-06 | 0.0298 | 0.0331 | 0.3683 |
| rs9410230 | T | C | -0.0276 | 0.0062 | 8.1369E-06 | -0.0975 | 0.0661 | 0.1401 |

Table S8.3 Variants Used as Instruments for Multivariable MR estimates for the causal effect of DR (adjusted for fasting glucose).

| **SNP** | **Effect Allele** | **Other Allele** | **Association with Fasting glucose** | | | **Association with Diabetic Retinopathy** | | |
| --- | --- | --- | --- | --- | --- | --- | --- | --- |
|  |  |  | **Beta** | **SE** | **P** | **Beta** | **SE** | **P** |
| rs10012946 | C | T | 0.0098 | 0.0021 | 4.30002E-06 | 0.0821 | 0.0332 | 0.0133601 |
| rs10061244 | G | A | -0.022 | 0.005 | 7.03008E-06 | -0.0246 | 0.0532 | 0.6432 |
| rs10203174 | T | C | -0.016 | 0.0036 | 8.67002E-06 | -0.1267 | 0.0684 | 0.0641505 |
| rs10811661 | C | T | -0.024 | 0.0028 | 5.64937E-18 | -0.0986 | 0.0466 | 0.0341397 |
| rs10814916 | C | A | 0.016 | 0.0022 | 2.25996E-13 | 0.0895 | 0.0328 | 0.00644199 |
| rs10830963 | G | C | 0.078 | 0.0025 | 1E-200 | 0.0195 | 0.0344 | 0.5712 |
| rs11195502 | T | C | -0.032 | 0.0037 | 1.97015E-18 | -0.0192 | 0.0579 | 0.740399 |
| rs11257655 | T | C | 0.013 | 0.0026 | 4.43997E-07 | 0.0627 | 0.0373 | 0.0925998 |
| rs11558471 | G | A | -0.029 | 0.0023 | 7.8001E-37 | -0.0386 | 0.0338 | 0.2534 |
| rs11603334 | A | G | -0.019 | 0.0028 | 1.11995E-11 | 0.0314 | 0.0385 | 0.4143 |
| rs11607883 | A | G | -0.021 | 0.0021 | 6.31975E-24 | -0.0702 | 0.0332 | 0.0347696 |
| rs11619319 | G | A | 0.02 | 0.0024 | 1.33015E-15 | -0.0408 | 0.0375 | 0.2762 |
| rs11672660 | T | C | -0.016 | 0.0028 | 5.83002E-09 | 0.0225 | 0.0377 | 0.551099 |
| rs11708067 | G | A | -0.023 | 0.0026 | 1.29987E-18 | -0.0484 | 0.044 | 0.2706 |
| rs11715915 | T | C | -0.012 | 0.0022 | 4.90004E-08 | -0.0493 | 0.0331 | 0.137 |
| rs12055786 | T | C | 0.011 | 0.0021 | 5.50998E-08 | -0.0095 | 0.0331 | 0.774499 |
| rs12423664 | A | G | 0.016 | 0.0033 | 1.10999E-06 | 0.009 | 0.0495 | 0.855 |
| rs1260326 | C | T | 0.029 | 0.0021 | 2.1702E-41 | 0.035 | 0.0344 | 0.3088 |
| rs1280 | C | T | -0.026 | 0.0031 | 8.56052E-18 | -0.0093 | 0.0482 | 0.847 |
| rs12888855 | A | C | -0.016 | 0.0025 | 5.03999E-10 | -0.0693 | 0.042 | 0.098951 |
| rs12908437 | C | T | -0.011 | 0.0022 | 6.32004E-07 | -0.0029 | 0.0343 | 0.9334 |
| rs16851397 | G | A | -0.027 | 0.005 | 6.64003E-08 | -0.0385 | 0.0892 | 0.665701 |
| rs16913693 | G | T | -0.043 | 0.0066 | 3.50994E-11 | -0.1142 | 0.1077 | 0.2889 |
| rs16970406 | C | T | 0.016 | 0.0035 | 0.00000751 | -0.0669 | 0.0565 | 0.2363 |
| rs17168486 | T | C | 0.031 | 0.0028 | 3.1703E-28 | 0.0299 | 0.0411 | 0.4663 |
| rs17331697 | C | T | -0.017 | 0.0036 | 0.00000137 | -0.1172 | 0.0555 | 0.03474 |
| rs174576 | A | C | -0.02 | 0.0022 | 1.18005E-18 | 0.0278 | 0.0333 | 0.4033 |
| rs17712208 | A | T | 0.051 | 0.0074 | 3.22033E-12 | -0.02 | 0.1244 | 0.8726 |
| rs1805081 | C | T | -0.0095 | 0.0021 | 5.72005E-06 | -0.0072 | 0.0331 | 0.8285 |
| rs194520 | G | T | 0.011 | 0.0022 | 1.72001E-06 | 0.0273 | 0.0332 | 0.4111 |
| rs1983127 | T | G | 0.011 | 0.0023 | 8.13992E-07 | -0.0794 | 0.0367 | 0.0305999 |
| rs2007854 | A | G | -0.03 | 0.0067 | 6.03004E-06 | -0.1362 | 0.1069 | 0.2027 |
| rs2053386 | T | C | -0.0095 | 0.0021 | 7.47997E-06 | -0.0489 | 0.0348 | 0.1595 |
| rs2191349 | T | G | 0.029 | 0.0021 | 1.27997E-42 | 0.0188 | 0.033 | 0.5689 |
| rs2476601 | G | A | 0.0094 | 0.0033 | 0.00462903 | -0.4128 | 0.047 | 1.6661E-18 |
| rs2657879 | G | A | 0.012 | 0.0027 | 5.68997E-06 | 0.0091 | 0.0424 | 0.8307 |
| rs267731 | G | A | -0.033 | 0.0073 | 4.60999E-06 | -0.0736 | 0.0907 | 0.4168 |
| rs2785137 | A | G | -0.011 | 0.0022 | 4.86004E-07 | 0.0448 | 0.035 | 0.2013 |
| rs314274 | C | A | -0.016 | 0.0034 | 3.92004E-06 | -0.0365 | 0.0351 | 0.2987 |
| rs3778321 | A | G | -0.014 | 0.0027 | 0.000000129 | -0.0155 | 0.0396 | 0.695799 |
| rs3801969 | T | G | 0.0095 | 0.0021 | 5.63002E-06 | 0.0565 | 0.0328 | 0.0850394 |
| rs3829109 | A | G | -0.017 | 0.0027 | 1.13E-10 | -0.0298 | 0.0344 | 0.3867 |
| rs4457053 | A | G | -0.012 | 0.0025 | 4.27996E-06 | 0.0201 | 0.0391 | 0.6081 |
| rs4502156 | C | T | -0.022 | 0.0021 | 1.38007E-25 | -0.0205 | 0.0332 | 0.5366 |
| rs459193 | G | A | 0.011 | 0.0023 | 1.58001E-06 | 0.0431 | 0.0353 | 0.2219 |
| rs4660698 | C | A | 0.012 | 0.0023 | 2.21998E-07 | -0.0172 | 0.0357 | 0.6294 |
| rs4869272 | T | C | 0.018 | 0.0022 | 1.02E-15 | -0.0286 | 0.0348 | 0.4104 |
| rs506597 | G | A | 0.015 | 0.0031 | 1.34999E-06 | 0.0882 | 0.0454 | 0.0520295 |
| rs560887 | C | T | 0.071 | 0.0025 | 1.3996E-178 | 0.0051 | 0.0357 | 0.8876 |
| rs577906 | A | G | -0.01 | 0.0022 | 4.83003E-06 | -0.0069 | 0.035 | 0.8442 |
| rs6072275 | A | G | 0.016 | 0.0028 | 1.66001E-08 | 0.0078 | 0.0384 | 0.8399 |
| rs6113722 | A | G | -0.035 | 0.0053 | 2.49E-11 | -0.1479 | 0.0876 | 0.0911696 |
| rs6733097 | A | C | -0.015 | 0.0033 | 4.79005E-06 | -0.0993 | 0.0459 | 0.0305302 |
| rs6752228 | T | G | 0.0091 | 0.002 | 8.10998E-06 | 0.0127 | 0.0329 | 0.6995 |
| rs6943153 | C | T | -0.015 | 0.0022 | 1.63005E-12 | 0.0284 | 0.0331 | 0.3907 |
| rs6975024 | C | T | 0.061 | 0.0029 | 2.8801E-99 | -0.0529 | 0.053 | 0.3186 |
| rs7178572 | G | A | 0.012 | 0.0022 | 1.63001E-07 | -0.0252 | 0.035 | 0.4705 |
| rs749067 | C | T | -0.017 | 0.0022 | 6.12068E-15 | -0.0286 | 0.0355 | 0.4201 |
| rs7651090 | G | A | 0.013 | 0.0023 | 1.75001E-08 | 0.0562 | 0.0354 | 0.1124 |
| rs7903146 | T | C | 0.022 | 0.0024 | 2.71019E-20 | 0.0779 | 0.0409 | 0.0569403 |
| rs8020333 | T | C | -0.011 | 0.0021 | 3.62001E-07 | 0.0275 | 0.0339 | 0.4164 |
| rs882020 | T | C | 0.021 | 0.003 | 3.04018E-12 | -0.0152 | 0.0439 | 0.729001 |
| rs983309 | G | T | -0.026 | 0.0033 | 6.29071E-15 | 0.0323 | 0.0464 | 0.4869 |

Table S8.4 Variants Used as Instruments for Multivariable MR estimates for the causal effect of non-proliferative DR (adjusted for HbA1C).

| **SNP** | **Effect Allele** | **Other Allele** | **Association with HbA1C** | | | **Association with Non-Proliferative Diabetic Retinopathy** | | |
| --- | --- | --- | --- | --- | --- | --- | --- | --- |
|  |  |  | **Beta** | **SE** | **P** | **Beta** | **SE** | **P** |
| rs1046896 | T | C | 0.0346 | 0.0032 | 1.57616E-26 | -0.0732 | 0.0768 | 0.341 |
| rs1387153 | T | C | 0.0258 | 0.0039 | 3.96096E-11 | -0.0558 | 0.0715 | 0.4348 |
| rs16926246 | T | C | -0.089 | 0.0057 | 3.11028E-54 | -0.1415 | 0.1319 | 0.2831 |
| rs1799884 | T | C | 0.038 | 0.0041 | 1.45111E-20 | -0.2348 | 0.1105 | 0.0336504 |
| rs2022059 | C | G | -0.007 | 0.0146 | 0.6326 | 1.2642 | 0.2071 | 1.02301E-09 |
| rs2779116 | T | C | 0.0237 | 0.004 | 2.751E-09 | 0.2039 | 0.0714 | 0.00430497 |
| rs4737009 | A | G | 0.0269 | 0.0039 | 6.11505E-12 | 0.0547 | 0.0783 | 0.4845 |
| rs552976 | G | A | 0.029 | 0.0034 | 8.15643E-18 | 0.0161 | 0.0714 | 0.8211 |
| rs6474359 | C | T | -0.0601 | 0.0105 | 1.17801E-08 | 0.0387 | 0.2593 | 0.8814 |
| rs7998202 | G | A | 0.0307 | 0.0053 | 5.23504E-09 | -0.0958 | 0.1025 | 0.3502 |
| rs855791 | G | A | -0.0271 | 0.0036 | 2.74221E-14 | -0.0315 | 0.0708 | 0.656 |
| rs9275373 | A | G | 0.0051 | 0.006 | 0.3952 | 1.4325 | 0.1116 | 1.08493E-37 |

Table S8.5 Variants Used as Instruments for Multivariable MR estimates for the causal effect of non-proliferative DR (adjusted for fasting glucose).

| **SNP** | **Effect Allele** | **Other Allele** | **Association with Fasting glucose** | | | **Association with Non-Proliferative Diabetic Retinopathy** | | |
| --- | --- | --- | --- | --- | --- | --- | --- | --- |
|  |  |  | **Beta** | **SE** | **P** | **Beta** | **SE** | **P** |
| rs10012946 | C | T | 0.0098 | 0.0021 | 4.3E-06 | 0.2163 | 0.0684 | 0.00156899 |
| rs10061244 | G | A | -0.022 | 0.005 | 7.0301E-06 | -0.1231 | 0.1091 | 0.2591 |
| rs10203174 | T | C | -0.016 | 0.0036 | 8.67E-06 | 0.0445 | 0.1394 | 0.749501 |
| rs10811661 | C | T | -0.024 | 0.0028 | 5.6494E-18 | -0.2115 | 0.0959 | 0.0273502 |
| rs10814916 | C | A | 0.016 | 0.0022 | 2.26E-13 | 0.1137 | 0.0675 | 0.0922104 |
| rs10830963 | G | C | 0.078 | 0.0025 | 1E-200 | -0.0358 | 0.0704 | 0.611299 |
| rs11195502 | T | C | -0.032 | 0.0037 | 1.9702E-18 | -0.0773 | 0.1196 | 0.5182 |
| rs11257655 | T | C | 0.013 | 0.0026 | 4.44E-07 | 0.0518 | 0.0767 | 0.4998 |
| rs11558471 | G | A | -0.029 | 0.0023 | 7.8001E-37 | 0.0092 | 0.0694 | 0.8942 |
| rs11603334 | A | G | -0.019 | 0.0028 | 1.12E-11 | 0.1148 | 0.0789 | 0.1459 |
| rs11607883 | A | G | -0.021 | 0.0021 | 6.3198E-24 | -0.0445 | 0.0682 | 0.5139 |
| rs11619319 | G | A | 0.02 | 0.0024 | 1.3302E-15 | -0.1378 | 0.0774 | 0.0749204 |
| rs11672660 | T | C | -0.016 | 0.0028 | 5.83E-09 | -0.0354 | 0.0772 | 0.6468 |
| rs11708067 | G | A | -0.023 | 0.0026 | 1.2999E-18 | -0.0121 | 0.0899 | 0.8933 |
| rs11715915 | T | C | -0.012 | 0.0022 | 4.9E-08 | -0.0022 | 0.0681 | 0.9745 |
| rs12055786 | T | C | 0.011 | 0.0021 | 5.51E-08 | -0.0382 | 0.0682 | 0.5754 |
| rs12423664 | A | G | 0.016 | 0.0033 | 1.11E-06 | -0.1741 | 0.1012 | 0.0851903 |
| rs1260326 | C | T | 0.029 | 0.0021 | 2.1702E-41 | 0.1173 | 0.0706 | 0.0966897 |
| rs1280 | C | T | -0.026 | 0.0031 | 8.5605E-18 | -0.0081 | 0.0992 | 0.9346 |
| rs12888855 | A | C | -0.016 | 0.0025 | 5.04E-10 | -0.0905 | 0.0867 | 0.2968 |
| rs12908437 | C | T | -0.011 | 0.0022 | 6.32E-07 | -0.0414 | 0.0705 | 0.557799 |
| rs16851397 | G | A | -0.027 | 0.005 | 6.64E-08 | 0.0078 | 0.1838 | 0.9661 |
| rs16913693 | G | T | -0.043 | 0.0066 | 3.5099E-11 | -0.4978 | 0.2246 | 0.02666 |
| rs16970406 | C | T | 0.016 | 0.0035 | 0.00000751 | -0.0757 | 0.118 | 0.5214 |
| rs17168486 | T | C | 0.031 | 0.0028 | 3.1703E-28 | -0.0236 | 0.0846 | 0.7804 |
| rs17331697 | C | T | -0.017 | 0.0036 | 0.00000137 | 0.0447 | 0.1136 | 0.6941 |
| rs174576 | A | C | -0.02 | 0.0022 | 1.1801E-18 | 0.1274 | 0.0686 | 0.0632004 |
| rs17712208 | A | T | 0.051 | 0.0074 | 3.2203E-12 | 0.5899 | 0.261 | 0.0238199 |
| rs1805081 | C | T | -0.0095 | 0.0021 | 5.7201E-06 | -0.0912 | 0.0678 | 0.1789 |
| rs194520 | G | T | 0.011 | 0.0022 | 1.72E-06 | 0.0933 | 0.068 | 0.1704 |
| rs1983127 | T | G | 0.011 | 0.0023 | 8.1399E-07 | -0.134 | 0.0754 | 0.0755892 |
| rs2007854 | A | G | -0.03 | 0.0067 | 6.03E-06 | -0.0531 | 0.2186 | 0.8082 |
| rs2053386 | T | C | -0.0095 | 0.0021 | 7.48E-06 | -0.0266 | 0.0714 | 0.7099 |
| rs2191349 | T | G | 0.029 | 0.0021 | 1.28E-42 | 0.0387 | 0.0677 | 0.567599 |
| rs267731 | G | A | -0.033 | 0.0073 | 4.61E-06 | 0.0915 | 0.1869 | 0.6243 |
| rs2785137 | A | G | -0.011 | 0.0022 | 4.86E-07 | 0.0851 | 0.0721 | 0.2377 |
| rs314274 | C | A | -0.016 | 0.0034 | 3.92E-06 | 0.1335 | 0.0727 | 0.0662293 |
| rs3778321 | A | G | -0.014 | 0.0027 | 1.29E-07 | 0.0878 | 0.0813 | 0.28 |
| rs3801969 | T | G | 0.0095 | 0.0021 | 5.63E-06 | 0.0342 | 0.0674 | 0.6122 |
| rs3829109 | A | G | -0.017 | 0.0027 | 1.13E-10 | -0.0261 | 0.0707 | 0.7119 |
| rs4457053 | A | G | -0.012 | 0.0025 | 4.28E-06 | -0.0396 | 0.0802 | 0.6214 |
| rs4502156 | C | T | -0.022 | 0.0021 | 1.3801E-25 | -0.0114 | 0.0682 | 0.8673 |
| rs459193 | G | A | 0.011 | 0.0023 | 1.58E-06 | 0.0501 | 0.0722 | 0.4882 |
| rs4660698 | C | A | 0.012 | 0.0023 | 2.22E-07 | 0.0023 | 0.0733 | 0.9749 |
| rs4869272 | T | C | 0.018 | 0.0022 | 1.02E-15 | 0.0331 | 0.0714 | 0.642899 |
| rs506597 | G | A | 0.015 | 0.0031 | 1.35E-06 | 0.0096 | 0.0934 | 0.9184 |
| rs560887 | C | T | 0.071 | 0.0025 | 1.4E-178 | 0.0171 | 0.0735 | 0.8158 |
| rs577906 | A | G | -0.01 | 0.0022 | 4.83E-06 | -0.1046 | 0.0718 | 0.1451 |
| rs6072275 | A | G | 0.016 | 0.0028 | 1.66E-08 | 0.0932 | 0.0787 | 0.2367 |
| rs6113722 | A | G | -0.035 | 0.0053 | 2.49E-11 | 0.3993 | 0.1785 | 0.0253198 |
| rs6679677 | A | C | -0.0092 | 0.0034 | 0.00609495 | 0.4646 | 0.096 | 0.00000129 |
| rs6733097 | A | C | -0.015 | 0.0033 | 4.7901E-06 | -0.0291 | 0.094 | 0.757201 |
| rs6752228 | T | G | 0.0091 | 0.002 | 8.11E-06 | 0.0561 | 0.0676 | 0.4066 |
| rs6943153 | C | T | -0.015 | 0.0022 | 1.6301E-12 | 0.1182 | 0.0678 | 0.0812194 |
| rs6975024 | C | T | 0.061 | 0.0029 | 2.88E-99 | -0.2252 | 0.1104 | 0.0412895 |
| rs7178572 | G | A | 0.012 | 0.0022 | 1.63E-07 | -0.1218 | 0.0718 | 0.0898008 |
| rs749067 | C | T | -0.017 | 0.0022 | 6.1207E-15 | -0.0532 | 0.0735 | 0.4687 |
| rs7583748 | A | G | -0.015 | 0.0034 | 0.00000426 | 0.0196 | 0.098 | 0.8413 |
| rs7651090 | G | A | 0.013 | 0.0023 | 1.75E-08 | 0.1046 | 0.0731 | 0.1526 |
| rs7903146 | T | C | 0.022 | 0.0024 | 2.7102E-20 | 0.1912 | 0.0842 | 0.0231499 |
| rs8020333 | T | C | -0.011 | 0.0021 | 3.62E-07 | -0.0604 | 0.0697 | 0.3868 |
| rs882020 | T | C | 0.021 | 0.003 | 3.0402E-12 | -0.0831 | 0.0896 | 0.3534 |
| rs9368222 | A | C | 0.014 | 0.0023 | 1E-09 | 0.1888 | 0.0716 | 0.00830807 |
| rs983309 | G | T | -0.026 | 0.0033 | 6.2907E-15 | 0.0415 | 0.0963 | 0.666099 |

Table S8.6 Variants Used as Instruments for Multivariable MR estimates for the causal effect of proliferative DR (adjusted for HbA1C).

| **SNP** | **Effect Allele** | **Other Allele** | **Association with HbA1C** | | | **Association with Proliferative Diabetic Retinopathy** | | |
| --- | --- | --- | --- | --- | --- | --- | --- | --- |
|  |  |  | **Beta** | **SE** | **P** | **Beta** | **SE** | **P** |
| rs10100688 | A | G | 0.0285 | 0.0057 | 4.82603E-07 | -0.017 | 0.0228 | 0.4558 |
| rs1046896 | T | C | 0.0346 | 0.0032 | 1.57616E-26 | 0.0143 | 0.0187 | 0.4434 |
| rs10506857 | G | C | -0.0157 | 0.0035 | 9.38901E-06 | -0.0109 | 0.0174 | 0.5324 |
| rs10806742 | C | T | -0.0215 | 0.0047 | 4.75401E-06 | 0.0459 | 0.0171 | 0.00723502 |
| rs10934830 | T | G | 0.0696 | 0.0154 | 6.00302E-06 | 0.0168 | 0.0374 | 0.6524 |
| rs11231694 | C | G | 0.035 | 0.0076 | 3.95103E-06 | -0.0035 | 0.0312 | 0.9098 |
| rs11243147 | T | C | 0.0009 | 0.0035 | 0.801 | 0.0921 | 0.0166 | 3.01801E-08 |
| rs11774990 | T | C | -0.0072 | 0.0049 | 0.1406 | 0.0919 | 0.0204 | 7.02894E-06 |
| rs11964178 | G | A | -0.0168 | 0.0034 | 8.78598E-07 | 0.0262 | 0.0164 | 0.1107 |
| rs12580246 | A | G | 0.0284 | 0.0061 | 0.000003755 | -0.028 | 0.0265 | 0.2908 |
| rs12819124 | A | C | -0.0161 | 0.0034 | 1.83101E-06 | -0.0069 | 0.0166 | 0.679301 |
| rs1387153 | T | C | 0.0258 | 0.0039 | 3.96096E-11 | 0.011 | 0.0174 | 0.528901 |
| rs16926246 | T | C | -0.089 | 0.0057 | 3.11028E-54 | -0.0276 | 0.032 | 0.3888 |
| rs16968859 | G | A | 0.0107 | 0.0089 | 0.2338 | -0.1097 | 0.0234 | 2.70097E-06 |
| rs17533945 | C | T | 0.0179 | 0.0038 | 1.93299E-06 | 0.0275 | 0.0165 | 0.0949992 |
| rs17789266 | T | C | 0.023 | 0.0047 | 8.13598E-07 | 0.0467 | 0.0272 | 0.0854398 |
| rs1789891 | A | C | -0.0207 | 0.0045 | 4.67498E-06 | 0.0132 | 0.0198 | 0.5039 |
| rs1799884 | T | C | 0.038 | 0.0041 | 1.45111E-20 | -0.0085 | 0.0266 | 0.7492 |
| rs2476601 | G | A | -0.0038 | 0.0057 | 0.5053 | -0.215 | 0.0231 | 1.50107E-20 |
| rs2523953 | A | G | 0.0068 | 0.0109 | 0.534399 | -0.1907 | 0.0262 | 3.60911E-13 |
| rs2597318 | G | A | -0.0006 | 0.0035 | 0.8536 | -0.0736 | 0.0166 | 9.79693E-06 |
| rs2723517 | C | A | -0.0159 | 0.0035 | 4.01698E-06 | 0.0104 | 0.0164 | 0.5279 |
| rs2779116 | T | C | 0.0237 | 0.004 | 2.751E-09 | 0.0216 | 0.0174 | 0.2129 |
| rs3006789 | T | C | 0.0168 | 0.0037 | 7.00197E-06 | -0.0085 | 0.0175 | 0.626 |
| rs3782123 | A | C | -0.0201 | 0.0042 | 1.67201E-06 | 0.0054 | 0.0191 | 0.7776 |
| rs3957146 | C | T | 0.0041 | 0.0061 | 0.509 | 0.8115 | 0.0262 | 3.9995E-211 |
| rs4502225 | C | T | -0.0113 | 0.0062 | 0.0673504 | -0.1201 | 0.0247 | 1.18899E-06 |
| rs4737009 | A | G | 0.0269 | 0.0039 | 6.11505E-12 | 0.0269 | 0.019 | 0.157 |
| rs4844390 | G | A | -0.0204 | 0.0041 | 6.89906E-07 | 0.0043 | 0.0198 | 0.8284 |
| rs552976 | G | A | 0.029 | 0.0034 | 8.15643E-18 | 0.0271 | 0.0173 | 0.1174 |
| rs6453220 | T | C | 0.0456 | 0.0099 | 4.18601E-06 | 0.0384 | 0.064 | 0.549 |
| rs6474359 | C | T | -0.0601 | 0.0105 | 1.17801E-08 | -0.1416 | 0.063 | 0.0245901 |
| rs6509507 | A | G | -0.0033 | 0.0081 | 0.6848 | 0.1022 | 0.0215 | 2.05698E-06 |
| rs6844670 | G | A | 0.0203 | 0.0039 | 2.123E-07 | -0.0031 | 0.0167 | 0.8546 |
| rs7260507 | C | A | -0.0042 | 0.0049 | 0.3854 | -0.0785 | 0.017 | 3.87802E-06 |
| rs7355559 | T | G | -0.0241 | 0.0052 | 3.86198E-06 | -0.0059 | 0.0289 | 0.8378 |
| rs7644261 | G | C | -0.0179 | 0.0037 | 1.45801E-06 | -0.0206 | 0.0185 | 0.2677 |
| rs7805376 | G | T | 0.0012 | 0.0035 | 0.7305 | -0.075 | 0.0167 | 7.34598E-06 |
| rs7805661 | C | A | 0.0366 | 0.0079 | 4.08997E-06 | 0.0841 | 0.0343 | 0.0141599 |
| rs7903146 | T | C | 0.0128 | 0.0034 | 0.000189099 | 0.1137 | 0.0205 | 2.81702E-08 |
| rs7998202 | G | A | 0.0307 | 0.0053 | 5.23504E-09 | 0.0094 | 0.025 | 0.707801 |
| rs8059028 | A | C | 0.0049 | 0.0038 | 0.1901 | 0.0777 | 0.0169 | 4.30002E-06 |
| rs837763 | T | C | 0.0245 | 0.0048 | 2.696E-07 | -0.0033 | 0.0165 | 0.8396 |
| rs855791 | G | A | -0.0271 | 0.0036 | 2.74221E-14 | -0.003 | 0.0172 | 0.8627 |
| rs915894 | G | T | 0.0031 | 0.0062 | 0.6158 | 0.3063 | 0.0185 | 1.1371E-61 |
| rs9318651 | T | C | 0.0367 | 0.0079 | 0.00000315 | -0.0024 | 0.0167 | 0.8856 |
| rs9354939 | C | T | -0.0163 | 0.0035 | 3.09999E-06 | 0.0015 | 0.0165 | 0.9269 |
| rs9394159 | T | A | 0.0054 | 0.0035 | 0.1155 | 0.0854 | 0.0164 | 1.911E-07 |
| rs9410230 | T | C | -0.0276 | 0.0062 | 8.13692E-06 | 0.0099 | 0.0332 | 0.764999 |

Table S8.7 Variants Used as Instruments for Multivariable MR estimates for the causal effect of proliferative DR (adjusted for fasting glucose).

| **SNP** | **Effect Allele** | **Other Allele** | **Association with Fasting glucose** | | | **Association with Proliferative Diabetic Retinopathy** | | |
| --- | --- | --- | --- | --- | --- | --- | --- | --- |
|  |  |  | **Beta** | **SE** | **P** | **Beta** | **SE** | **P** |
| rs10012946 | C | T | 0.0098 | 0.0021 | 4.30002E-06 | 0.0487 | 0.0166 | 0.00338602 |
| rs10061244 | G | A | -0.022 | 0.005 | 7.03008E-06 | -0.0203 | 0.0267 | 0.4474 |
| rs10203174 | T | C | -0.016 | 0.0036 | 8.67002E-06 | -0.0779 | 0.0341 | 0.0224999 |
| rs10811661 | C | T | -0.024 | 0.0028 | 5.64937E-18 | -0.0554 | 0.0234 | 0.0177799 |
| rs10814916 | C | A | 0.016 | 0.0022 | 2.25996E-13 | 0.0319 | 0.0164 | 0.0521495 |
| rs10830963 | G | C | 0.078 | 0.0025 | 1E-200 | 0.0076 | 0.0172 | 0.657099 |
| rs11195502 | T | C | -0.032 | 0.0037 | 1.97015E-18 | -0.0152 | 0.029 | 0.6008 |
| rs11257655 | T | C | 0.013 | 0.0026 | 4.43997E-07 | 0.0194 | 0.0186 | 0.2994 |
| rs11558471 | G | A | -0.029 | 0.0023 | 7.8001E-37 | -0.0293 | 0.0169 | 0.0829106 |
| rs11603334 | A | G | -0.019 | 0.0028 | 1.11995E-11 | -0.0363 | 0.0193 | 0.0603295 |
| rs11607883 | A | G | -0.021 | 0.0021 | 6.31975E-24 | -0.0459 | 0.0167 | 0.005863 |
| rs11619319 | G | A | 0.02 | 0.0024 | 1.33015E-15 | -0.0143 | 0.0188 | 0.4467 |
| rs11672660 | T | C | -0.016 | 0.0028 | 5.83002E-09 | -0.0018 | 0.0188 | 0.9225 |
| rs11708067 | G | A | -0.023 | 0.0026 | 1.29987E-18 | -0.0277 | 0.0219 | 0.2066 |
| rs11715915 | T | C | -0.012 | 0.0022 | 4.90004E-08 | -0.0291 | 0.0166 | 0.0791699 |
| rs12055786 | T | C | 0.011 | 0.0021 | 5.50998E-08 | 0.0061 | 0.0165 | 0.7122 |
| rs12423664 | A | G | 0.016 | 0.0033 | 1.10999E-06 | -0.026 | 0.0247 | 0.2923 |
| rs1260326 | C | T | 0.029 | 0.0021 | 2.1702E-41 | 0.0263 | 0.0172 | 0.1266 |
| rs1280 | C | T | -0.026 | 0.0031 | 8.56052E-18 | -0.0039 | 0.0242 | 0.8724 |
| rs12888855 | A | C | -0.016 | 0.0025 | 5.03999E-10 | -0.0036 | 0.021 | 0.8645 |
| rs12908437 | C | T | -0.011 | 0.0022 | 6.32004E-07 | -0.0108 | 0.0172 | 0.528 |
| rs16851397 | G | A | -0.027 | 0.005 | 6.64003E-08 | -0.0052 | 0.0449 | 0.9078 |
| rs16913693 | G | T | -0.043 | 0.0066 | 3.50994E-11 | 0.0341 | 0.0536 | 0.5239 |
| rs17168486 | T | C | 0.031 | 0.0028 | 3.1703E-28 | 0.0046 | 0.0206 | 0.8247 |
| rs17331697 | C | T | -0.017 | 0.0036 | 0.00000137 | -0.0199 | 0.0276 | 0.4709 |
| rs174576 | A | C | -0.02 | 0.0022 | 1.18005E-18 | 0.0084 | 0.0166 | 0.6134 |
| rs17712208 | A | T | 0.051 | 0.0074 | 3.22033E-12 | 0.1342 | 0.0619 | 0.0302099 |
| rs1805081 | C | T | -0.0095 | 0.0021 | 5.72005E-06 | 0.0011 | 0.0165 | 0.9451 |
| rs194520 | G | T | 0.011 | 0.0022 | 1.72001E-06 | 0.0324 | 0.0166 | 0.0512802 |
| rs1983127 | T | G | 0.011 | 0.0023 | 8.13992E-07 | -0.0362 | 0.0183 | 0.0479303 |
| rs2007854 | A | G | -0.03 | 0.0067 | 6.03004E-06 | -0.0296 | 0.0535 | 0.580301 |
| rs2053386 | T | C | -0.0095 | 0.0021 | 7.47997E-06 | -0.0046 | 0.0174 | 0.7896 |
| rs2191349 | T | G | 0.029 | 0.0021 | 1.27997E-42 | 0.0224 | 0.0165 | 0.1732 |
| rs2476601 | G | A | 0.0094 | 0.0033 | 0.00462903 | -0.215 | 0.0231 | 1.50107E-20 |
| rs2657879 | G | A | 0.012 | 0.0027 | 5.68997E-06 | 0.0062 | 0.0212 | 0.7689 |
| rs267731 | G | A | -0.033 | 0.0073 | 4.60999E-06 | -0.0042 | 0.0454 | 0.926 |
| rs2785137 | A | G | -0.011 | 0.0022 | 4.86004E-07 | 0.0192 | 0.0175 | 0.2747 |
| rs314274 | C | A | -0.016 | 0.0034 | 3.92004E-06 | -0.0309 | 0.0176 | 0.0784603 |
| rs3801969 | T | G | 0.0095 | 0.0021 | 5.63002E-06 | 0.037 | 0.0164 | 0.0242399 |
| rs3829109 | A | G | -0.017 | 0.0027 | 1.13E-10 | -0.0295 | 0.0172 | 0.0870302 |
| rs4457053 | A | G | -0.012 | 0.0025 | 4.27996E-06 | 0.0052 | 0.0196 | 0.791099 |
| rs4502156 | C | T | -0.022 | 0.0021 | 1.38007E-25 | -0.0043 | 0.0166 | 0.7944 |
| rs459193 | G | A | 0.011 | 0.0023 | 1.58001E-06 | 0.0369 | 0.0176 | 0.0361102 |
| rs4660698 | C | A | 0.012 | 0.0023 | 2.21998E-07 | 0.0166 | 0.0178 | 0.3512 |
| rs4869272 | T | C | 0.018 | 0.0022 | 1.02E-15 | -0.0063 | 0.0174 | 0.719201 |
| rs506597 | G | A | 0.015 | 0.0031 | 1.34999E-06 | 0.0543 | 0.0228 | 0.0169801 |
| rs560887 | C | T | 0.071 | 0.0025 | 1.3996E-178 | 0.0144 | 0.0179 | 0.4222 |
| rs577906 | A | G | -0.01 | 0.0022 | 4.83003E-06 | -0.0281 | 0.0175 | 0.1076 |
| rs6072275 | A | G | 0.016 | 0.0028 | 1.66001E-08 | 0.0583 | 0.0192 | 0.00244101 |
| rs6113722 | A | G | -0.035 | 0.0053 | 2.49E-11 | -0.0394 | 0.0435 | 0.3658 |
| rs6733097 | A | C | -0.015 | 0.0033 | 4.79005E-06 | -0.042 | 0.023 | 0.0675601 |
| rs6752228 | T | G | 0.0091 | 0.002 | 8.10998E-06 | -0.0162 | 0.0165 | 0.3241 |
| rs6943153 | C | T | -0.015 | 0.0022 | 1.63005E-12 | -0.0015 | 0.0165 | 0.9262 |
| rs6975024 | C | T | 0.061 | 0.0029 | 2.8801E-99 | -0.0096 | 0.0266 | 0.717999 |
| rs7178572 | G | A | 0.012 | 0.0022 | 1.63001E-07 | 0.0188 | 0.0175 | 0.2845 |
| rs749067 | C | T | -0.017 | 0.0022 | 6.12068E-15 | -0.0217 | 0.0177 | 0.2208 |
| rs7583748 | A | G | -0.015 | 0.0034 | 0.00000426 | 0.0138 | 0.0238 | 0.561 |
| rs7651090 | G | A | 0.013 | 0.0023 | 1.75001E-08 | 0.0397 | 0.0177 | 0.0251501 |
| rs7903146 | T | C | 0.022 | 0.0024 | 2.71019E-20 | 0.1137 | 0.0205 | 2.81702E-08 |
| rs8020333 | T | C | -0.011 | 0.0021 | 3.62001E-07 | -0.0057 | 0.017 | 0.7356 |
| rs8059028 | A | C | 0.004 | 0.0022 | 0.0672822 | 0.0777 | 0.0169 | 4.30002E-06 |
| rs882020 | T | C | 0.021 | 0.003 | 3.04018E-12 | -0.0188 | 0.0219 | 0.3925 |
| rs9394159 | T | A | 0.0015 | 0.0021 | 0.455113 | 0.0854 | 0.0164 | 1.911E-07 |
| rs983309 | G | T | -0.026 | 0.0033 | 6.29071E-15 | 0.0116 | 0.0232 | 0.6167 |
